# Ecdysone signaling mediates the trade-off between immunity and reproduction via suppression of amyloids in the mosquito *Aedes aegypti*

**DOI:** 10.1371/journal.ppat.1010837

**Published:** 2022-09-22

**Authors:** Mao Wang, Yanhong Wang, Mengmeng Chang, Xueli Wang, Zuokun Shi, Alexander S. Raikhel, Zhen Zou

**Affiliations:** 1 State Key Laboratory of Integrated Management of Pest Insects and Rodents, Institute of Zoology, Chinese Academy of Sciences, Beijing, People’s Republic of China; 2 CAS Center for Excellence in Biotic Interactions, University of Chinese Academy of Sciences, Beijing, People’s Republic of China; 3 Department of Entomology and Institute for Integrative Genome Biology, University of California, Riverside, California, United States of America; Institut Pasteur, FRANCE

## Abstract

The balance between immunity and reproduction is essential for many key physiological functions. We report that to maintain an optimal fertility, 20-hydroxyecdysone (20E) and the ecdysone receptor (EcR) downregulate the immune deficiency (IMD) pathway during the post blood meal phase (PBM) of the *Aedes aegypti* reproductive cycle. RNA interference-mediated depletion of EcR elicited an increased expression of the IMD pathway components, and these mosquitoes were more resistant to infection by Gram-negative bacteria. Moreover, 20E and EcR recruit Pirk-like, the mosquito ortholog of *Drosophila melanogaster* Pirk. CRISPR-Cas9 knockout of Pirk-like has shown that it represses the IMD pathway by interfering with IMD-mediated formation of amyloid aggregates. 20E and EcR disruption of the amyloid formation is pivotal for maintaining normal yolk protein production and fertility. Additionally, 20E and its receptor EcR directly induce Pirk-like to interfere with cRHIM-mediated formation of amyloid. Our study highlights the vital role of 20E in governing the trade-off between immunity and reproduction. Pirk-like might be a potential target for new methods to control mosquito reproduction and pathogen transmission.

## Author summary

To reproduce, hematophagous female mosquitoes require repeated blood feeding that consequentially makes them crucial vectors of many devastating diseases. A steroid hormone 20-hydroxyecdysone (20E) plays a critical role in mosquito reproduction. It is also essential for maintaining a balance between reproduction and immunity. Following a blood meal, the ecdysone receptor (EcR) directly induces an inhibitor Pirk-like, affecting the immune deficiency (IMD) pathway. Pirk-like downregulates the IMD pathway by disrupting the amyloid formation and preventing the nuclear translocation of the NF-κB transcriptional factor Rel2. The EcR downregulation of the immune response and the amyloid formation is vital for maintaining the mosquito fertility. This study identified the Pirk-like–amyloid immune axis that could be a target for new mosquito control methods.

## Introduction

Hematophagous female mosquitoes utilize vertebrate blood as their source of nutrition and energy for egg production. Blood feeding on humans allows them to transmit virus, *Plasmodium*, and nematode pathogens. In blood-feeding mosquitoes, gonadotrophic cycle consists of a post-eclosion phase (PE) in the first cycle and a post blood meal phase (PBM) that are regulated by two major insect hormones, juvenile hormone (JH) and 20-hydroxyecdysone (20E), respectively [1]. JH controls preparatory previtellogenic events, while 20E is the principal hormone regulating vitellogenesis, egg maturation and ovulation [2]. 20E binds to the heterodimeric receptor complex of the ecdysone receptor (EcR) and Ultraspiracle (USP) [3,4], and initiates the transcriptional cascade responsible for the expression of vitellogenin (Vg) genes [5,6]. Vg proteins are secreted from the fat body and subsequently taken up by oocytes for egg maturation [1]. The fat body tissue is the central site for both vitellogenesis and immune defense [7].

Insect humoral immunity is composed of two evolutionarily conserved NF-κB pathways, Toll and immune deficiency (IMD), which lead to the production of antimicrobial peptides (AMPs) in response to peptidoglycan (PGN) infection from bacteria [8–10]. Diaminopimelic acid-containing PGN specifically triggers the IMD pathway by its most proximal components—peptidoglycan recognition proteins (PGRP-LC or PGRP-LE) [11]. There is a conserved sequence motif in the N-terminal domains of PGRP-LC and PGRP-LE [12] that has weak homology to the mammalian receptor interacting protein (RIP) homotypic interaction motifs (RHIM) and is referred to as a cryptic RHIM (cRHIM) in *Drosophila melanogaster*. The cRHIMs in PGRP-LC and PGRP-LE form amyloid fibrils and trigger the IMD pathway [13]. As a result, the downstream NF-κB transcriptional factor Relish (Rel2) is translocated to the nucleus and initiates the transcription of multiple immune effectors targeting invading pathogens [14,15].

Immunity and reproduction are highly demanding processes and, thus, a balance must be maintained between the two. Reduced reproductive output and capacity have been observed after a bacterial challenge or the immune signaling activation in *D*. *melanogaster* [16,17]. In septically injured *Anopheles* mosquitoes, apoptosis of follicular cells occurs, resulting in a reduced oviposition [18–20]. Conversely, the increased reproductive output can downregulate the constitutive and inductive immunity of female insects. For example, mating leads to a reduced survival rate of *D*. *melanogaster* females in response to infection with various pathogens, with an observed higher pathogen load and decreased AMP expression level [21,22]. Mating reduces cellular encapsulation and melanization in crickets *Acheta domesticus* and *Allonemobius socius* [23–25]. These important observations inspired us to decipher the molecular interactions between reproduction and immunity in *Aedes aegypti*, a mosquito vector of numerous human viral diseases.

The pleiotropic effects of JH and 20E make them prime candidates for controlling the allocation of resources among different physiological processes [26,27]. In *D*. *melanogaster*, mated females are more likely to die from septic injury and have less ability to induce an immune response. These defects are rescued when JH signaling is suppressed [28]. JH downregulates the expression of immunity-related genes (IMRGs) in *Ae*. *aegypti* during the PE stage [29]. However, the role of 20E in the trade-off between reproduction and immunity remains elusive in *Ae*. *aegypti*. Here, we have identified that 20E and EcR in *Ae*. *aegypti* downregulate the IMD pathway to protect reproductive output. Moreover, we provide evidence that, for this action, the 20E-EcR-USP complex directly activates the expression of Pirk-like, a negative regulator of the IMD pathway [30]. Our study highlights that the regulatory role of 20E in the IMD pathway is crucial for mosquito fertility, which may further affect the coordination of innate immune and reproductive responses.

## Results

### Ecdysone signaling downregulates the IMD pathway during the PBM reproductive phase

**Fig 1:**
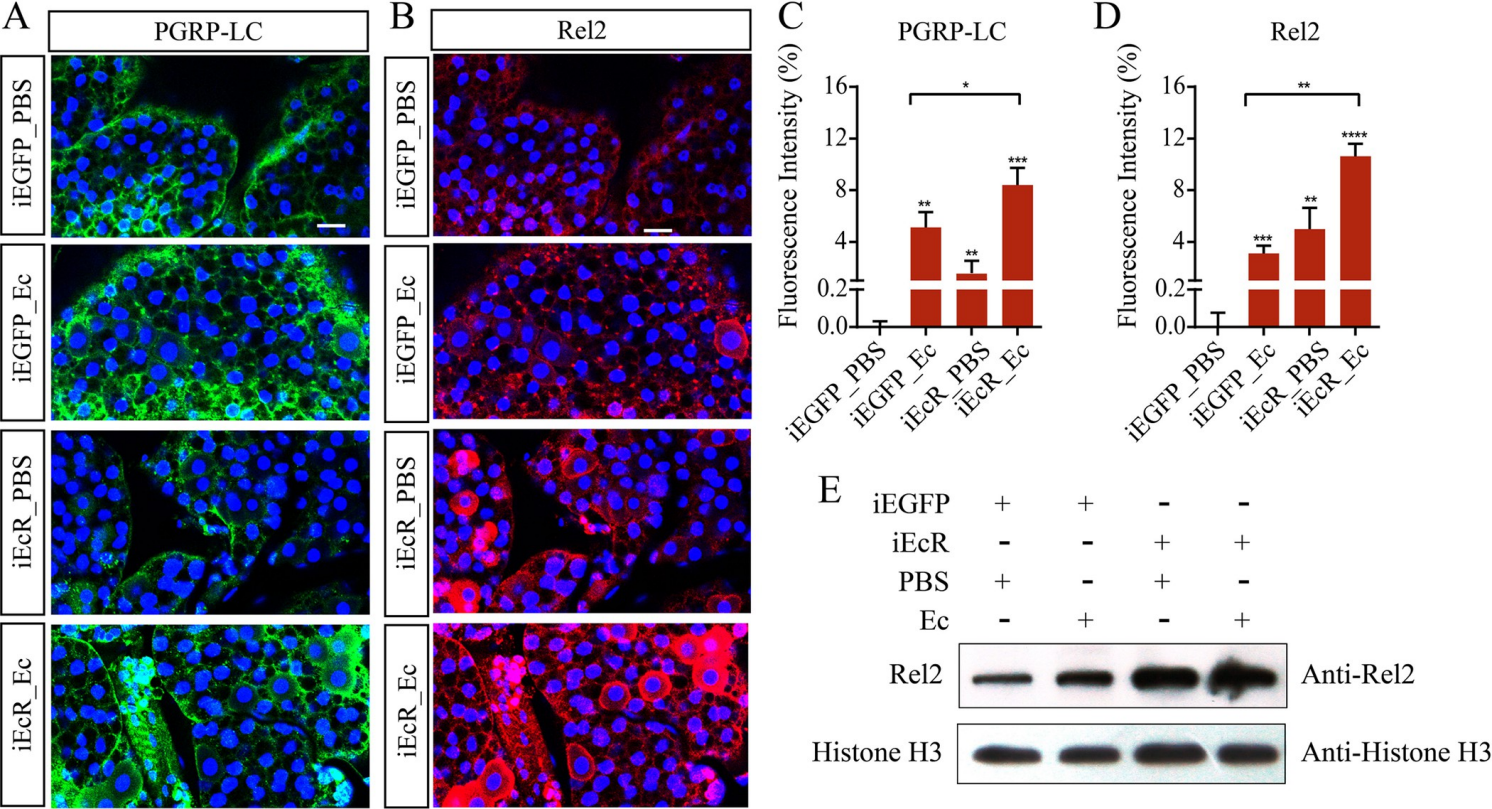
RNAi silencing of EcR results in elevated activation of PGRP-LC and Rel2 in response to bacterial infection. (A and B) Immunofluorescence of PGRP-LC (A) and Rel2 (B) in iEGFP and iEcR mosquito fat bodies, stimulated for 12 h with PBS or *E*. *cloacae* 12 h after blood meal. Scale bar, 20 μm. (C and D) Quantification of relative fluorescence intensity of cells stained using PGRP-LC or Rel2 antibodies. (E) Protein levels of Rel2 in the nucleus of iEGFP and iEcR mosquitoes that were either non-infected or infected with *E*. *cloacae*. For each group, nuclear protein extracts were performed from extracts of fat bodies from ten female mosquitoes after 24 h of *E*. *cloacae* challenge. Histone H3 were used as controls. Data are from three biological replicates.

The role of 20E in mosquito reproduction has been characterized in great detail [31,32]. Following a blood meal, a 20E pulse [33] initiates massive gene expression [34]. However, the role of 20E in immunity during the *Ae*. *aegypti* PBM reproductive phase has received little attention. To explore the interplay between the 20E and IMD pathway, mosquitoes were injected with EcR dsRNA (iEcR) to block 20E signaling at 2 days PE and then infected with Gram-negative bacteria *Enterobacter cloacae* at 12 h PBM. The fat bodies were dissected for immunofluorescence analysis at 24 h PBM, a time when both the immune response and the 20E titer are high. The knockdown efficiency of EcR was verified (S1A Fig). The iEcR_Ec mosquitoes demonstrated a higher *PGRP-LC* expression than iEGFP_Ec mosquitoes (S1B Fig). An immunofluorescence staining assay also revealed that PGRP-LC was significantly induced in iEcR_PBS mosquitoes relative to the iEGFP_PBS females. The iEcR mosquitoes exhibited a considerably higher expression of PGRP-LC after *E*. *cloacae* infection (Fig 1A and 1C).

**Fig 2:**
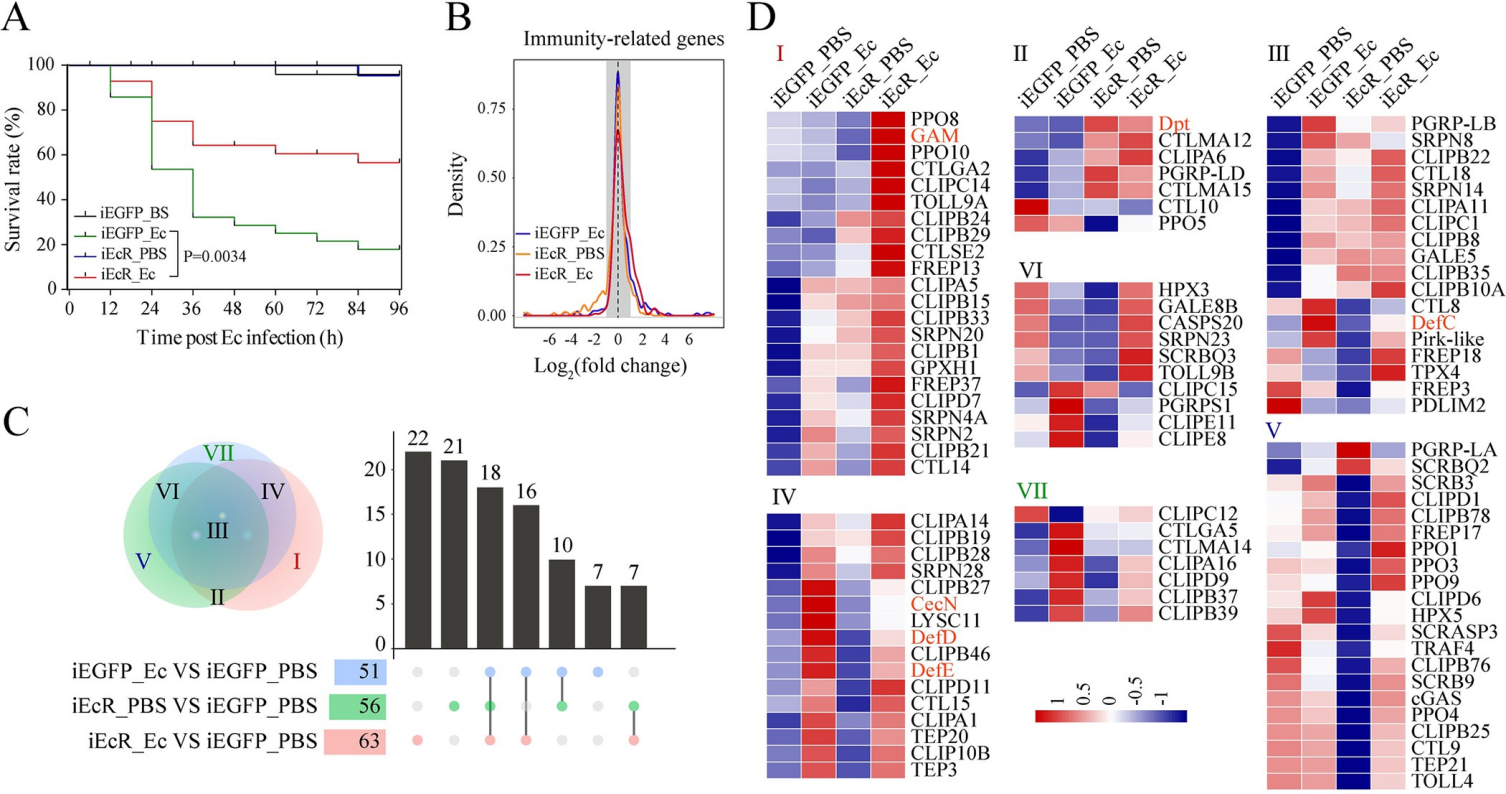
Silencing of EcR increases susceptibility to infection and induces IMRG expression. (A) Survival of EcR knockdown (iEcR) or control (iEGFP) mosquitoes after *E*. *cloacae* infection. n = 3 cohorts (total 90 mosquitoes). P values for differences in survival times between iEGFP and iEcR mosquitoes were determined by means of the log-rank test. (B) Density plot showing the effect of EcR silencing (iEcR) on IMRG expression after *E*. *cloacae* or PBS treatment. Gray shaded area indicates values used to define differentially expressed genes or resistant genes. (C) Venn diagram and UpSet plot representing unique and shared IMRGs. The overlapping regions represent gene cohorts that are concomitantly regulated by one or two experimental conditions. (D) Hierarchical cluster analysis of IMRGs in (C) with log_2_(FPKM) values. Gene names are shown on the right. Data are from three biological replicates. Pearson correlation >0.97 for all replicates.

Next, we analyzed the effect of EcR depletion on the expression of Rel2, the mosquito ortholog of Drosophila Relish, during the PBM phase. Immunofluorescence showed a strong Rel2 abundance in iEcR mosquito fat bodies treated with PBS, which was significantly increased at 12 h post infection with *E*. *cloacae* (Fig 1B and 1D), consistent with mRNA abundance (S1C Fig). After a similar experiment was performed in iEcR mosquitoes at 24 h post infection, an even higher increase in Rel2 abundance was observed, with the majority of the Rel2 signal accumulated in the nuclei. The latter was confirmed using western blots (Figs 1E and S1D–S1E). Furthermore, the survival rate after *E*. *cloacae* infection was significantly higher in iEcR mosquitoes than in iEGFP_Ec mosquitoes (Fig 2A). These data strongly suggest that 20E and EcR negatively regulate the immune response in mosquitoes.

To gain further insight into the effect of 20E signaling on expression of IMRGs, we performed RNA-sequencing (RNA-Seq) of iEcR mosquito fat bodies after a blood meal, infected with *E*. *cloacae* or PBS for 12 h. A set of IMRGs was expressed at a significantly higher level in iEcR_Ec than iEGFP_Ec mosquitoes (Fig 2B). We also analyzed the shared and unique differentially expressed IMRGs among three treatment samples (iEcR_Ec, iEcR_PBS and iEGFP_Ec) and compared them with the non-infected control sample (iEGFP_PBS). Identified IMRGs were classified into seven groups (Fig 2C). Among these genes, 58 were highly activated in iEcR_Ec mosquitoes and only moderately activated in iEGFP_Ec mosquitoes, relative to the iEGFP_PBS control. Many of these highly activated genes were identified as components of the IMD pathway, AMPs, melanization, and the Toll pathway (Fig 2D).

Interestingly, transcriptional induction of *Dpt*, C-type lectin (*CTLMA12* and *CTLMA15*) and a variety of clip-domain serine proteases was also observed in iEcR_PBS fat bodies (Figs 2D and S2), suggesting that 20E signaling downregulates the expression of the major components of innate immunity under naive conditions.

### Pirk-like responds to 20E at the PBM reproductive phase

**Fig 3:**
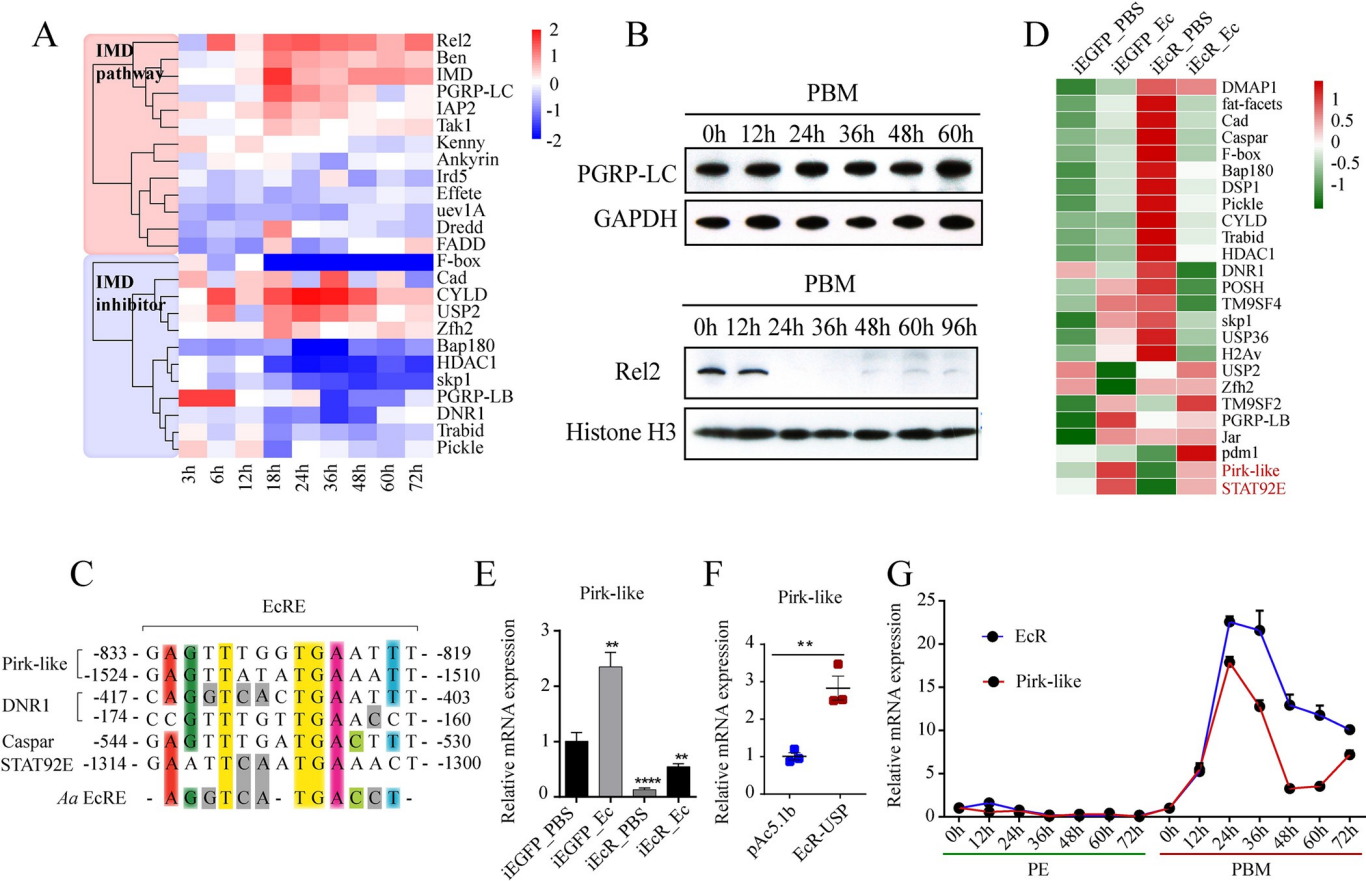
Pirk-like expression is regulated by 20E signaling. (A) Heat map of transcriptional patterns of the IMD pathway component genes during PBM phases. Gene expression at each time point was normalized to PE 72 h. (B) Protein levels of PGRP-LC and Rel2 determined using western blots. For each time point, both cytoplasmic and nuclear protein extracts were prepared from ten female mosquito fat bodies. GAPDH and Histone H3 were used as controls. (C) Putative EcRE of the IMD pathway inhibitors aligned with reported *Aedes* EcRE sequences. The three core amino acids are highlighted with yellow. (D) Heatmap showing the expression pattern of 25 IMD inhibitor genes in iEcR mosquitoes compared with iEGFP mosquitoes upon *E*. *cloacae* infection. (E) mRNA abundance of *Pirk-like* in iEcR mosquitoes infected by *E*. *cloacae*. Bar plots are shown as mean ± SEM. ****p < 0.0001 (one-way ANOVA followed by Bartlett’s test). Data are from three biological replicates. (F) mRNA abundance of *Pirk-like* in Aag2 cells co-transfected EcR and USP. (G) Expression of Pirk-like and EcR in the PE and PBM phases. Dot plots represent mean ± SEM. Data are from three biological replicates.

To identify potential factors of the IMD pathway responding to 20E signaling, we first analyzed the overall expression of genes involved in the IMD pathway using custom-made Agilent microarray chips at nine time points (3–72 h) during the PBM phase [34,35]. Hierarchical clustering analysis revealed that *PGRP-LC*, *IMD*, *Ben*, *IAP2* and *Rel2* with upregulated expressions were positively correlated with 20E titer (Fig 3A). However, we found that the protein level of PGRP-LC was consistent during the PBM phase, and Rel2 was barely detected in the nucleus from 24 h to 60 h PBM (Fig 3B). Therefore, the inhibitors of the IMD pathway might respond to 20E signaling. Through sequence alignment, we found 29 reported IMD inhibitors in *Ae*. *aegypti*. Among them, an ortholog of *D*. *melanogaster* Pirk (29% similarity) was identified. It has 105 amino acid residues and does not contain any functional domain or cRHIM motif; thus, it was designated as Pirk-like (S3A and S3B Fig).

Jaspar program analysis revealed that the promoter regions of *Pirk-like*, *DNR1*, *STAT92E* and *Caspar* contain highly conserved EcRE (Fig 3C), whereas only *Pirk-like* was significantly decreased in non-infected iEcR mosquitoes (Fig 3D and 3E). In addition, *Pirk-like* increased in iEcR mosquitoes after septic injury, but was lower than in infected iEGFP mosquitoes, suggesting that *Pirk-like* might be co-regulated by 20E and immune stimulation in the PBM phase. Moreover, *Pirk-like* was induced by 20E in EcR- and USP-overexpressed *Ae*. *aegypti* (Aag2) cells (Fig 3F). The time course of *Pirk-like* expression showed that *Pirk-like* was very low throughout the PE phase, but significantly increased during the PBM phase, with an expression peak coincident with that of *EcR* (Fig 3G). Pirk-like appears to be regulated by 20E to inhibit the IMD pathway during the PBM phase.

### The 20E-EcR-USP complex binds to the regulatory region of the *pirk-like* gene

**Fig 4:**
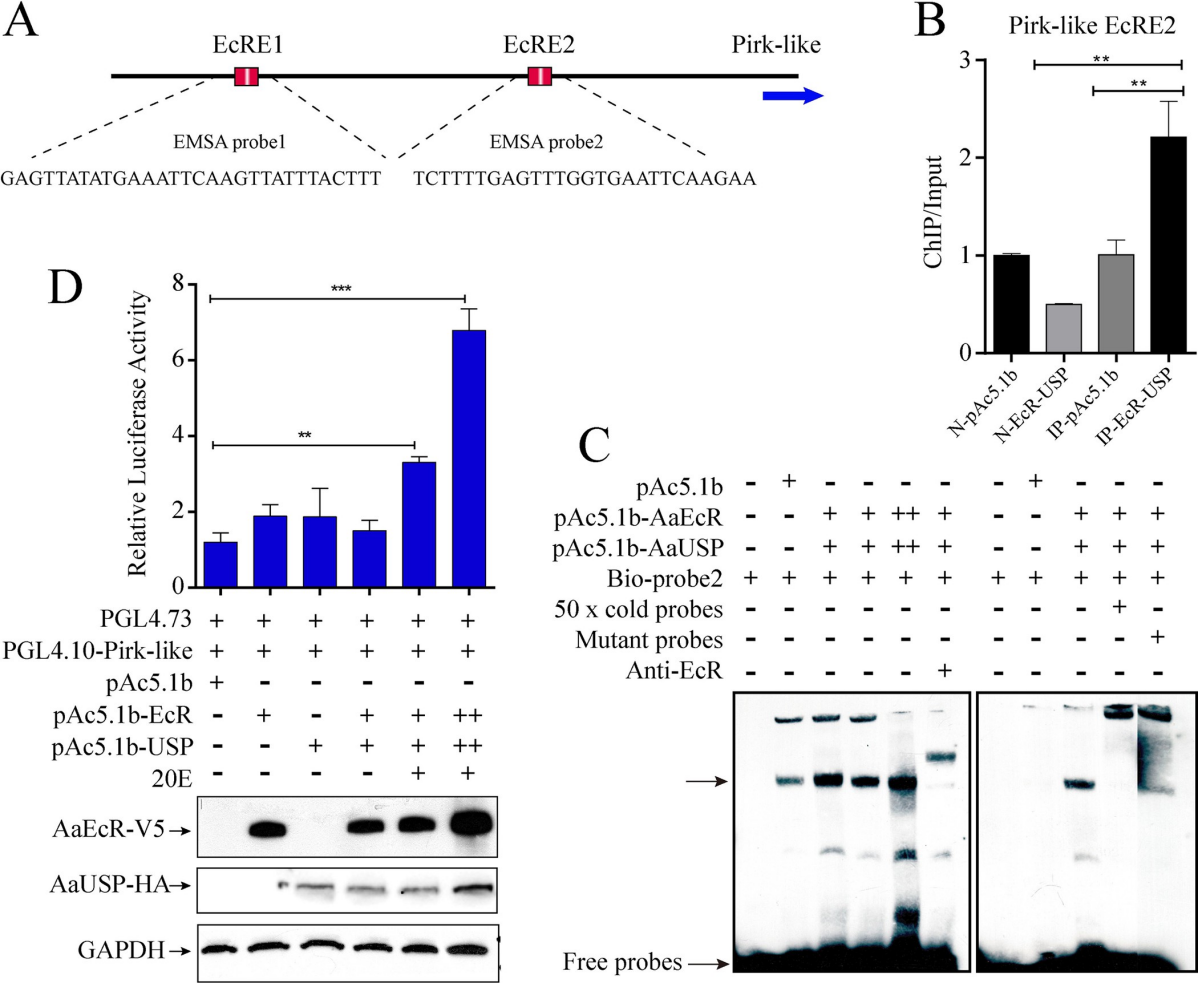
20E-EcR-USP directly binds to the EcRE motif of Pirk-like. (A) Schematic showing *Pirk-like* promoter and putative 20E-EcR-USP binding motifs. EcRE1 and EcRE2 represent regional target sites tested in ChIP-qPCR analysis. (B) ChIP-qPCR analysis with EcR and USP co-expressed Aag2 cells using V5 antibody. pAc5.1b lysates incubated with mouse IgG antibody (N-pAc5.1b) or anti-V5 (IP-pAc5.1b) and EcR-USP lysates incubated with mouse IgG antibody (N-EcR-USP) were included as controls. n = 3 biological replicates. (C) EMSA results showing the binding of EcR to a biotin-labeled EcRE2 probe. Nuclear protein extracts from EcR- and USP-overexpressed Aag2 cells. Competitor (unlabeled 26-bp probe), mutant competitor and anti-EcR antibody were added as indicated. (D) Dual-luciferase reporter assay revealed the transcriptional activation of *Pirk-like* promoter in EcR- and USP-overexpressed S2 cells with 20E treatment. S2 cells were co-transfected with the reporter vector pGL4.10-Pirk (100 ng), the overexpression vectors (pAc5.1b-EcR/V5 and pAc5.1b-USP/HA, 100 ng), and the pGL4.73 plasmid (15 ng). The relative luciferase activity was detected at 48 h after transfection. Western blots were used to analyze the protein level of EcR and USP using V5 and HA antibodies. GAPDH was used as the control.

The induction of *Pirk-like* in response to the 20E pulse in the PBM phase prompted us to identify whether *Pirk-like* is a direct target of 20E and its receptor EcR. We identified two conserved EcRE motifs located at -1590 bp and -818 bp in the *Pirk-like* gene regulatory region (Figs 3C and 4A). To assess the binding interaction of the *Pirk-like* gene promoter with EcR, we conducted chromatin immunoprecipitation (ChIP) coupled with quantitative polymerase chain reaction (qPCR). ChIP signals were enriched at the EcRE2 of *Pirk-like* in EcR- and USP-expressed cells (Fig 4B). The enrichment of EcRE1 was barely detected.

Next, we performed an electrophoretic gel mobility assay (EMSA) to analyze the interaction between EcR-USP and *Pirk-like*. The addition of Aag2 lysates containing co-expressed V5-EcR and V5-USP fusion proteins produced clear shifts of two biotin-labeled oligonucleotide fragments, derived from the EcRE1 and EcRE2 of *Pirk-like* (Figs 4C and S4). Control (pAc5.1b) lysates showed a slight binding band, which could come from endogenous EcR and USP in Aag2 cells. The specific interaction of protein and DNA was confirmed by a competitor with the unlabeled specific probe. More importantly, the shift after pre-incubating the nuclear extract with anti-EcR antibody was higher than others (Figs 4C and S4). This indicated that the binding of the anti-EcR antibodies and the antigen (V5-EcR) interfered with the migration of the probes. The EcRE2 mutant competitor considerably reduced the intensity of the specific band biotin-labeled *Pirk-like* probe bound to the V5-EcR-USP complex (Fig 4C). This verified the functionality of the EcRE2 of *Pirk-like*, confirming direct binding between EcR-USP and the *Pirk-like* promoter *in vitro*.

In addition, we performed a dual-luciferase reporter assay to study the interaction between EcR-USP and the *Pirk-like* promoter *in vivo*. Co-expressed V5-EcR and HA-USP resulted in a 5.6-fold increased induction of the *Pirk-like*-luciferase reporter after 20E treatment (Fig 4D), and showed the ability of EcR and USP to induce the *Pirk-like* expression in a dose-dependent manner. These data demonstrate that the 20E-EcR-USP heterodimer directly binds to the EcRE2 of *Pirk-like*, mediating its expression in the presence of 20E.

### Pirk-like interacts with and disrupts the formation of amyloid aggregates

**Fig 5:**
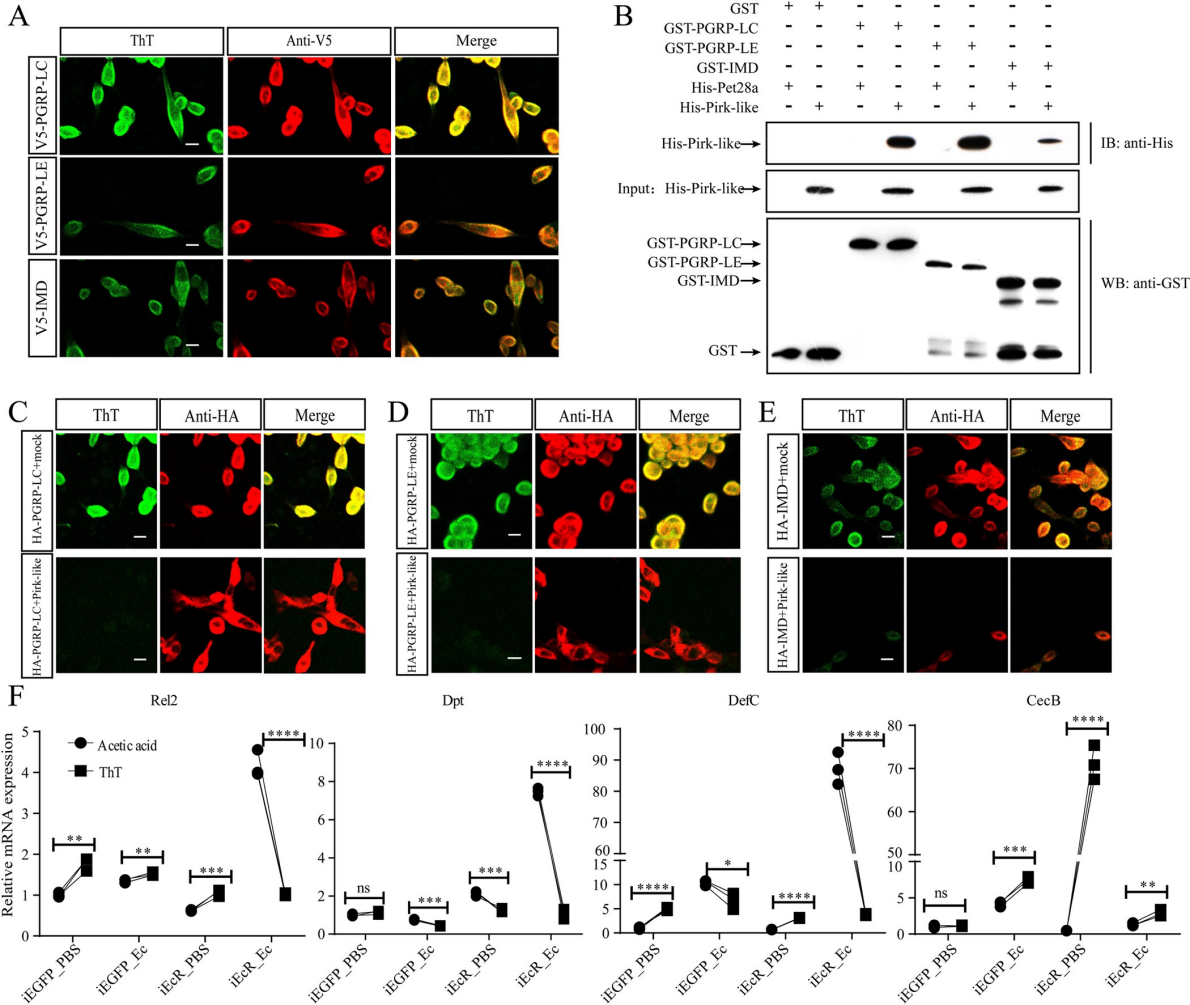
Pirk-like interferes with amyloidal aggregates formed by PGRP-LC, PGRP-LE, and IMD. (A) Amyloidal aggregate staining of Aag2 cells transiently transfected with V5-tagged PGRP-LC, PGRP-LE or IMD. Scale bar: 10 μm. (B) GST pull-down assay between PGRP-LC, PGRP-LE, IMD, and Pirk-like. The interactions of His-Pirk-like with GST-PGRP-LC, GST-PGRP-LE or GST-IMD were observed in the pull-down assay and confirmed using western blots with anti-GST and anti-His antibodies. (C-E) ThT fluorescence in Aag2 cells co-expressed with Ha-tagged PGRP-LC (C), PGRP-LE (D) or IMD (E), and either an empty vector (mock) or a V5-Pirk-like. Scale bar: 10 μm. (F) Expression of *Rel2*, *Dpt*, *DefC* and *CecB* in iEGFP and iEcR mosquitoes infected with *E*. *cloacae* in the presence of ThT. Acetic acid was used as control. Data are shown as mean ± SEM. ****p < 0.0001; ns, not significant (one-way ANOVA followed by Bartlett’s test).

The core motif IQIG or VQVG of mammalian RHIMs fold into cross-β sheet conformations and shape functional amyloid fibrils, which is related to necrosis signaling [36–38]. In *D*. *melanogaster*, the cRHIM of PGRP-LC, PGRP-LE, and IMD can form amyloid fibrils [13,39]. We found that *Ae*. *aegypti* PGRP-LE and IMD have the VHIG motif, a cRHIM in *D*. *melanogaster* [13], and that the cRHIMs of PGRP-LC are highly conserved with mammalian RHIMs, having the third position (P3) V substituted to N (S5A Fig). This sequence similarity suggested that amyloidal aggregates could be formed in *Ae*. *aegypti*. Thus, we used thioflavin T (ThT) fluorescence to measure amyloids in Aag2 cells. The cells with these overexpressed factors of V5-tagged PGRP-LC, PGRP-LE or IMD showed strong ThT fluorescence (Fig 5A). These results demonstrate that *Ae*. *aegypti* cRHIM can form amyloid fibrils, despite the sequence variability.

In *D*. *melanogaster*, Pirk directly interacts with PGRP-LC, PGRP-LE, and IMD via cRHIM [13]. Since cRHIM was not present in *Ae*. *aegypti* Pirk-like (S3A Fig), we wanted to investigate whether Pirk-like interacts with these proteins. The yeast two-hybrid (Y2H) assay showed that Pirk-like could bind to PGRP-LE (S5B–S5D Fig). PGRP-LC and IMD were self-activated when co-transformed with pGBKT7 (S5D Fig). Furthermore, a pull-down assay identified the presence of His-tagged-Pirk-like (His-Pirk-like) in the PGRP-LC (GST-PGRP-LC), PGRP-LE (GST-PGRP-LE), and IMD (GST-IMD) immunoprecipitation, confirming their interaction *in vitro* (Fig 5B).

We then determined whether Pirk-like could affect the IMD signaling by blocking amyloid formation. To test this, V5-tagged Pirk-like was co-transfected along with HA-tagged PGRP-LC, PGRP-LE, or IMD into Aag2 cells. Formaldehyde-fixed cells were stained with ThT, and the presence of the amyloidal aggregates was determined using confocal microscopy. The expression of PGRP-LC, PGRP-LE, and IMD, as detected by HA antibody staining, was similar in both mock and Pirk-like co-transfected cells. However, ThT signaling was extremely weak in Pirk-like co-expressed cells (Fig 5C–5E), indicating that Pirk-like terminates the formation of amyloids in Aag2 cells. Collectively, Pirk-like interacts with and suppresses the amyloid formation from PGRP-LC, PGRP-LE, and IMD.

In the next step, we examined the effect of EcR silencing on cRHIMs-mediated amyloid formation in fat bodies at PBM 24 h. EcR was translocated into the nuclei of iEGFP_PBS and iEGFP_Ec fat bodies with little detectable ThT signals. However, the dsRNA silencing of EcR significantly promoted amyloid formation, with or without *E*. *cloacae* infection (S5E Fig). ThT not only binds to amyloid fibrils but also inhibits further aggregation of amyloid [40]. We therefore postulated that ThT could rescue the expression of AMPs in iEcR mosquitoes. To examine this, iEcR mosquitoes were injected with ThT at 12 h PBM and then infected with *E*. *cloacae*. ThT caused a noticeable decrease in mRNA abundance of *Rel2*, *Dpt*, and *DefC* in infected iEcR mosquitoes, except for *CecB* (Fig 5F). ThT also reduced the expression of *Dpt* and *DefC* in infected iEGFP mosquitoes. In contrast, the mRNA level of *CecB* was strongly increased after ThT injection. These data re-affirm that 20E inhibits the IMD pathway by modulating the formation of amyloid and indicate that ThT specifically inhibits the IMD pathway.

### Pirk-like deficient mosquitoes exhibit a hyper-immune response and enhanced resistance to bacteria

**Fig 6:**
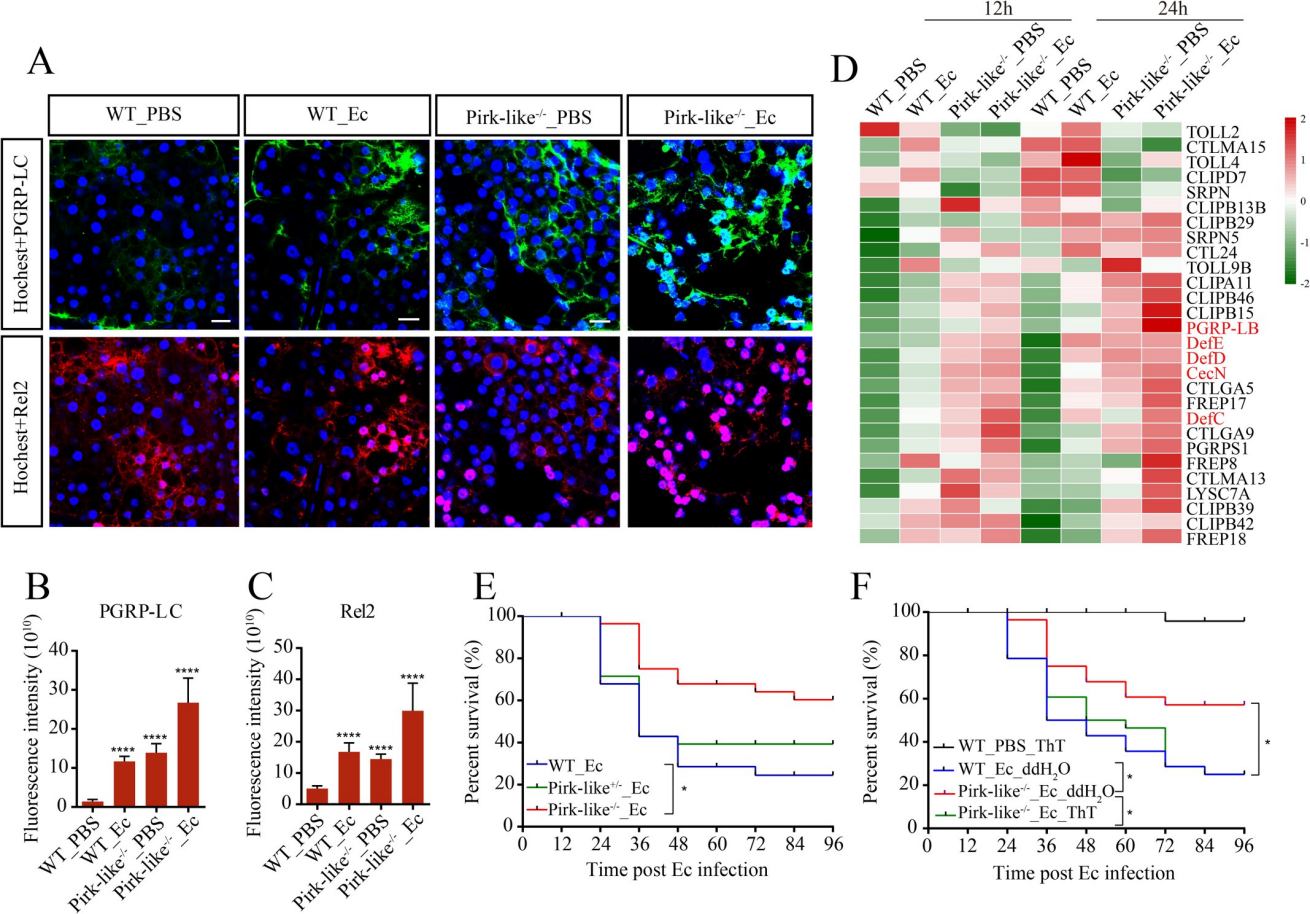
Pirk-like acts as an essential negative regulator of the IMD pathway. (A) Immunostaining of WT and Pirk-like^-/-^ mosquito fat bodies that were stimulated with PBS or *E*. *cloacae* at 12 h PBM and then dissected to measure PGRP-LC and Rel2 expressions at 24 h post infection. Scale bar: 20 μm. (B and C) Quantification of relative fluorescence intensity of cells shown in (A). Quantification showing mean ± SEM from three independent experiments. (D) Heatmap of the 28 shared IMRGs. They show the highest abundance among bacteria-infected Pirk-like^-/-^ mosquitoes (Pirk-like^-/-^_Ec) and the lowest among bacteria-infected WT mosquitoes (WT_Ec), with an intermediate level of abundance in non-infected Pirk-like^-/-^ mosquitoes (Pirk-like^-/-^_PBS). Non-infected WT mosquitoes (WT_PBS) were used as control. (E) Survival rate of Pirk-like^-/-^, Pirk-like^+/-^ and WT female mosquitoes after *E*. *cloacae* infection. n = 3 cohorts (total 90 mosquitoes). (F) Survival rate of Pirk-like^-/-^ and WT female mosquitoes after infection. Mosquitoes were fed with 1 mM ThT during the PBM phase. n = 3 cohorts (total 90 mosquitoes).

To better characterize the physiological function of Pirk-like, we generated Pirk-like deficient mosquitoes (Pirk-like^-/-^) using CRISPR-Cas9. Fat body immunohistochemistry showed that Pirk-like^-/-^ mosquitoes induced much higher expression of PGRP-LC and Rel2 after infection with *E*. *cloacae* (Pirk-like^-/-^_Ec), and Rel2 began to accumulate in the nucleus. Pirk-like^-/-^ mosquitoes treated with PBS (Pirk-like^-/-^_PBS) also showed strong PGRP-LC and Rel2 abundances, similar to those of infected wild type (WT_Ec) mosquitoes (Fig 6A–6C), suggesting that Pirk-like knockout sufficiently activates the IMD pathway after a blood meal.

To investigate the Pirk-like function in more detail, we conducted RNA-Seq of WT and Pirk-like^-/-^ fat bodies stimulated with PBS or *E*. *cloacae* after a blood meal. About 2000 genes were suppressed by Pirk-like. A density plot showed the effect of Pirk-like knockout on IMRG expression (S6A Fig). We observed that IMRGs were highly upregulated in Pirk-like^-/-^ mosquitoes, especially in samples treated with PBS or *E*. *cloacae* for 12 h. Using gene set enrichment analysis of differentially expressed IMRGs, we identified 28 IMRGs closely related to Pirk-like deficiency through four datasets (S6B Fig). Pirk-like^-/-^_Ec mosquitoes had a higher anti-bacterial response characterized by elevated transcription of a set of C-type lectins, clip-domain serine proteases, and AMPs in fat bodies than WT_Ec mosquitoes or normal controls (WT_PBS; Figs 6D and S6C–S6E). *DefD*, *DefC*, *DefE*, *Rel2*, *CTLGA5*, and *FREP18* were strongly induced in non-infected Pirk-like^-/-^ mosquitoes (Figs 6D and S6D). This over-activation of IMRGs in Pirk-like^-/-^_PBS mosquitoes implies that WT mosquitoes elicit a robust expression of Pirk-like to suppress the activation of innate immunity after a blood meal.

The survival rate of Pirk-like^-/-^ mosquitoes increased after septic injury (Fig 6E) and was rescued after feeding with ThT (Fig 6F), indicating that ThT effectively terminates activation of the IMD pathway and rescues the phenotype of Pirk-like^-/-^ mosquitoes. We also confirmed the feedback role of Pirk-like in the IMD pathway with iRel2 mosquitoes after *E*. *cloacae* infection. *E*. *cloacae* infection triggered drastic *Pirk-like* expression in iEGFP mosquitoes in both PE and PBM phases. Silencing Rel2 significantly downregulated *Pirk-like* expression on *E*. *cloacae* infection (S6F Fig), indicating that *Pirk-like* is likely a direct transcriptional target of Rel2. During PE phase, only the transcript of *CecB* was elevated in Pirk-like^-/-^ mosquitoes after PBS treatment, and the mRNA abundance of *CecB*, *CecG*, *DefC*, *DefD* and *GAM* was significantly higher in Pirk-like mosquitoes than in WT mosquitoes after infection (S6I Fig). These data show that Pirk-like is an important negative feedback regulator of the IMD pathway and that its absence leads to an exaggerated immune response that enables faster bacterial clearance, especially in PBM phase.

### Activation of the IMD pathway impairs reproductive output

**Fig 7:**
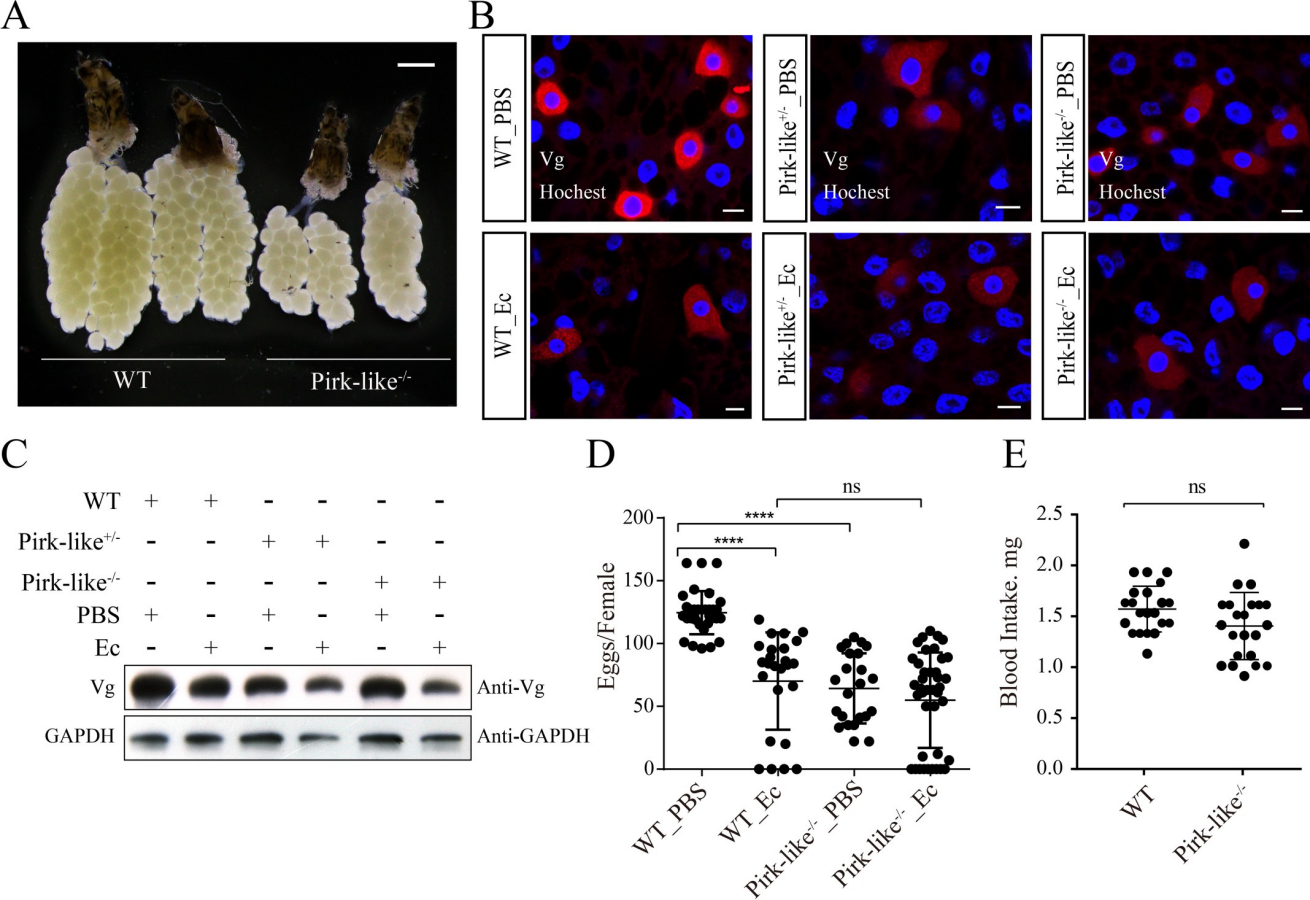
Knockout of Pirk-like impairs fecundity of mosquitoes. (A) Ovary development of WT and Pirk-like^-/-^ mosquitoes at 36 h PBM. Data represent at least three biological replicates. (B) Immunofluorescence of Vg expression in WT, Pirk-like^+/-^ and Pirk-like^-/-^ mosquitoes was observed using a confocal microscope. Scale bar: 20 μm. (C) WT, Pirk-like^+/-^ and Pirk-like^-/-^ mosquitoes either uninfected or infected with *E*. *cloacae* for 12 h were analyzed by means of western blots with Vg antibody. (D) Egg numbers of Pirk-like^-/-^ and WT female mosquitoes that were stimulated with PBS or *E*. *cloacae* (OD600 = 0.8) at 12 h PBM. Data represent three biological replicates with three technical replicates and are shown as mean ± SEM. (E) Blood intake of female mosquitoes at the time after a blood feeding (0 h PBM).

The over-activation of the immune system in insects in response to microbes can have a negative impact on female reproduction by reducing the accumulation of nutritive protein in ovaries, and by causing apoptosis of follicular cells and a decline in egg production [27,41]. This could allow for prioritization of limited resource utilization toward immunity and recovery from infection [42,43]. The IMD pathway is activated in Pirk-like^-/-^ mosquitoes in the PBM phase (Figs 6A and S6G), making Pirk-like^-/-^ mosquitoes a convenient model to study the fecundity cost associated with immune activation. Gene set enrichment analysis (GSEA) of RNA-seq datasets revealed that most of the genes involved in FoxO and oocyte meiosis pathways were suppressed in Pirk-like^-/-^_PBS 12 h mosquitoes (S6H and S7A and S7B Figs). This suggests that egg development might be abnormal in Pirk-like^-/-^ mosquitoes. We analyzed ovarian development in Pirk-like^-/-^ and WT mosquitoes and found that Pirk-like^-/-^ females failed to mature ovaries fully. Some females had one side ovary completely underdeveloped (Fig 7A). Ovary development shows no difference between WT and Pirk-like^-/-^ mosquitoes in PE phase (S7C Fig).

Fluorescence staining showed that Vg was strongly expressed in WT female fat bodies at 24 h PBM. In contrast, treatment with *E*. *cloacae* significantly reduced Vg levels in WT mosquitoes, indicating that immune activation impaired Vg expression. This was also observed in both Pirk-like^-/-^ and Pirk-like^+/-^ mosquitoes. This may be a cost of the IMD pathway activation, especially after septic injury (Fig 7B and 7C). *Vg* expression in Pirk-like^+/-^ and Pirk-like^-/-^ mosquitoes was strikingly decreased (S7D Fig) and there was a reduced deposition of Vg in ovaries (S7E Fig). Accordingly, Pirk-like^-/-^_PBS females laid fewer eggs than WT_PBS females, similar with WT_Ec mosquitoes (Fig 7D). Similarly, egg production was lower in iPirk-like females than iEGFP mosquitoes (S7F Fig). The differences in their egg productions could be due to different amounts of blood intake. Thus, we measured female weights, before and after blood-meals, and calculated their blood intake. Results showed that Pirk-like^-/-^ and WT mosquitoes had similar amounts of blood intake (Fig 7E). The egg production of iEGFP_Ec and iEcR_Ec mosquitoes was significantly reduced compared to control sample (S7G Fig). Accordingly, reduced Vg level in iEcR_Ec mosquitoes was observed (S7H Fig). These data indicate that activation of the IMD pathway impairs *Ae*. *aegypti* reproduction.

## Discussion

The trade-off between immunity and reproduction in many organisms suggests a common underlying mechanism associated with hormonal signaling [27]. JH, acting through its receptor methoprene-tolerant, suppresses IMRGs in *Ae*. *aegypti* during the PE reproductive phase [29]. In this study, we revealed the restrictive action of 20E on the bacteria-induced activation of the IMD pathway during the PBM reproductive phase of *Ae*. *aegypti*. Mechanistically, the ortholog of *D*. *melanogaster* Pirk, designated as Pirk-like, was upregulated by 20E and its receptor EcR in the fat body, after which it prevented the initiation of IMD signaling by interacting with PGRP-LC, PGRP-LE, and IMD. Our data also show that Pirk-like knockout mosquitoes are highly resistant to bacterial infection, and their reproductive output is significantly affected.

20E might directly induce certain immune effectors, allowing female mosquitoes to defend mild infection. This was supported by the finding that *PPO5*, *Toll4*, *Toll9B*, and *CTL10* expression was reduced in the absence of EcR (S2A–S2B Fig). However, when facing severe pathogenic bacteria infection, the mosquitoes failed to mobilize the IMD pathway on time, resulting in death (Fig 2A). A study in *D*. *melanogaster* revealed that 20E-triggered *PGRP-LC* expression controlled all IMD pathway outputs [44]. We identified 20E as a negative regulator of the IMD pathway in the mosquito PBM phase. The difference observed between these two species could be due to the dependency of mosquito reproduction on a blood meal, which may crosstalk with other factors such as FOXO, HR3, and E93 [32,45].

During the PBM phase, 20E and its receptor EcR activate transcriptional cascades regulating vitellogenesis [34] and lipid [46] and carbohydrate metabolism [47] to aid female reproduction. Simultaneously, 20E blocks certain physiological processes from occurring prematurely. It has been shown that autophagy, which is essential for the termination of vitellogenic events in the fat body at 36–42 h PBM, is inhibited by 20E at the peak of vitellogenesis, 18–24 h PBM [32]. We found that 20E suppressed the IMD pathway by inducing Pirk-like, following a blood meal (Fig 3G), and that the 20E-EcR-USP complex can directly regulate the expression of *Pirk-like* by binding to its regulatory region (Fig 4).

In mammals, the core RHIM motifs of RIPK1 and RIPK3 alternately stack into a hetero-amyloid and form cross-β sheets. Two of these sheets combine through hydrophobic interactions, and the hydrophobic I and V are tightly packed in the structure [38]. Residues outside the hydrophobic core, such as Q and N, form hydrogen bonds along the axis of the fibrils to stabilize the amyloid structure [38]. *Ae*. *aegypti* cRHIMs are different from mammalian RHIMs; only PGRP-LC has the amyloidogenic Q residue in the cRHIM core. But the V and G in the cRHIM core of PGRP-LC, PGRP-LE, and IMD are absolutely conserved with RHIMs (S5A Fig), suggesting that the insect cRHIMs might form a similar structure to mammalian RHIMs. Importantly, we propose that Pirk-like is sufficient to abolish amyloid formation without the cRHIM motif. In addition to the IMD pathway, Pirk-like also downregulates genes in ribosome, lysosome, synaptic vesicle cycle, and amino sugar and nucleotide sugar metabolism pathways (S6G Fig).

In adult insects, immunity and reproduction compete for limited resources. The direct physiological conflict between these two processes produces trade-offs, and individuals can partition limited energies, as needed, from one process to the other [27]. For example, increased reproductive activity reduces cellular encapsulation and melanization in *wood ant queens* [23,48]. Decreased AMP gene expression and lytic activity have been observed in *D*. *melanogaster* [22], and bacterial or fungal infection has been shown to reduce egg production in many insects [17,24]. Our results demonstrated that abolishing of Pirk-like enhanced mosquito resistance to bacteria; this was indicated by a hyper-activation of IMRGs and increased survival rate. However, Pirk-like^-/-^ mosquitoes suffered decreased *Vg* expression coupled with impaired development of ovaries and reduced egg productions. Thus, Pirk-like might become a potential target for new methods to control insect reproduction and pathogen infection.

Finally, we argued that hormonal signaling is crucial for regulating immune reactions and reproduction, and more broadly for governing homeostasis between distinct physiological processes. In *D*. *melanogaster*, JH antagonistically regulates reproduction, and innate immunity is thought to be the trade-off between these two processes [27]. In *Tenebrio molitor*, mating reduces phenoloxidase activity in both sexes, and this reduction is mediated by JH [49]. In mosquitoes, dramatic physiological events are triggered following a blood meal, including digestion and absorption of blood, excretion, coordination of high levels of gene expression, and rapid egg maturation [50]. Mosquitoes must develop specific adaptive capabilities to maintain homeostasis of their energy requirements during reproduction. We found that *PGRP-LC* and *IMD* were elevated after a blood meal. However, the increased transcriptional level was offset by 20E-induced Pirk-like because the activation of the IMD pathway can impair female mosquito reproduction. This study highlights the pleiotropic roles of 20E in maintaining mosquito homeostasis, which integrates nutrition, immunity, and reproduction signals to maximize the reproductive output.

## Methods

### Ethics statement

All procedures for using vertebrate animals were approved by the Animal Care and Use Committee of the Institute of Zoology at the Chinese Academy of Sciences (Approval: IOZ20190061).

### Animals

*Ae*. *aegypti* (Liverpool strain) mosquitoes were raised in the laboratory in an incubator at 28°C and 65% relative humidity using standard rearing procedures [51]. Adults were fed with 10% sucrose solution and water. Female mosquitoes were fed with chicken blood to initiate reproduction.

### Bacterial culture and septic injury

Bacterial *E*. *cloacae* strain was cultivated on LB plates or LB medium without antibiotics at 28°C and 80% relative humidity. Unless indicated, *E*. *cloacae* (OD600 = 1) was used in septic injury as described previously [52].

### Cell lines

Aag2 cells were cultivated at 27°C in Schneider’s *Drosophila* medium (Gibco) with 8% heat-inactivated fetal bovine serum (BSA, Invitrogen) for maintenance, transfection, and septic injury. *D*. *melanogaster* S2 cells were grown in SFX-INSECT (HyClone) with no antibiotics at 27°C and routinely passaged at 80% confluency.

### RNA extraction, cDNA synthesis and quantitative reverse-transcription PCR

Fat body tissues were lysed using TRIzol reagent (Invitrogen), and total RNA was extracted according to a protocol described previously [50]. Then, single-stranded cDNA was synthesized using PrimeScript RT reagent Kit with gDNA Erase (TaKaRa) starting from 1 μg total RNA. The qRT-PCR of target genes was performed using SuperReal PreMix (Tiangen) in a StepOnePlus Fast Real-Time PCR system (Thermo Fisher Scientific), and each reaction was run in triplicate. Template concentration was normalized to an endogenous control *ribosomal protein S7* (*RPS7*). The primers used for qRT-PCR are listed in the S1 Table.

### RNA interference experiments

The dsRNA was generated and purified using the T7 RiboMAX Express RNAi system (Promega) following the manufacturer’s protocol. Briefly, a 1-μg sample of dsRNA was microinjected into the thorax of female mosquitoes using a Nanoliter 2000 injector (World Precision Instrument). The EGFP gene was used as control dsRNA. After 3 days of recovery, mosquitoes were fed with chicken blood and then challenged with *E*. *cloacae* at 12 h PBM. Total RNA was collected from fat bodies at 12 h or 24 h after *E*. *cloacae* infection, and this was followed by qRT-PCR analysis. The primers used in the RNAi are listed in the S1 Table.

### Survival rate analysis

After 3 days, the females injected with dsRNA were fed with chicken blood and, 12 h later, infected with *E*. *cloacae*. The survival (30 mosquitoes/group) was counted every 12 h for 4 days after infection. Survival rates were compared using GraphPad 6.0. The Log-rank test was used to calculate the p value, and p < 0.05 was considered statistically significant.

### Ecdysone response elements (EcRE) predictions

We identified the promoter region of 40 genes in the IMD pathway using the Bio Mart tool of the VectorBase website (https://vectorbase.org/vectorbase/app). Then, combined with the previously reported EcRE of *Ae*. *Aegypti* [53], JASPAR (http://jaspar.genereg.net/) and Transfac were used [54] to predict the EcRE in the promoter region of these genes.

### Dual-luciferase reporter assay

The selected promoter region of *Pirk-like* (1307 bp, PGL4.10-Pirk-like) was cloned in PGL4.10 luciferase reporter vector. The overexpression vectors were constructed by insertion of the ORF of *EcR* and *USP* into the V5 and HA-tagged pAc5.1b vectors, respectively (pAc5.1b-EcR and -USP). Before transfection, S2 cells were seeded in a 24-well plate containing 400 μL medium per well and cultured for 2 h. Then, 100 ng of reporter and 100 ng of overexpression vectors were co-transfected with 15 ng of PGL4.73 Renilla luciferase vector using Cellfectin II Reagent (Gibco). After 40 h, the transfected cells were treated with 20E for 8 h and then processed with the dual-luciferase reporter assay system (Promega) to measure the expression of firefly luciferase and Renilla luciferase, following manufacturer’s instructions. Data were normalized to the *Renilla* luciferase activity.

### Electrophoretic mobility shift assay (EMSA)

Aag2 cells were transfected with the vectors pAc5.1b, pAc5.1b-EcR and -USP using FuGENE 6 Transfection Reagent (Promega). After 40 h, the transfected cells were treated with 20E for another 8 h to perform EMSAs as described previously [46]. Oligonucleotides used in EMSAs are listed in the S1 Table.

### Yeast two-hybrid analysis

Yeastmaker Yeast Transformation System 2 (Clontech) was used to perform Y2H analysis. The *Ae*. *aegypti* DNA sequence encoding Pirk-like was cloned into the pGBKT7 vector (BD-Pirk-like), and PGRP-LC, PGRP-LE, and IMD were cloned into the pGADT7 vector (AD-PGRP-LC, AD-PGRP-LE, and AD-IMD). The plasmids AD-PGRP-LC, AD-PGRP-LE, and AD-IMD were co-transformed with BD-Pirk-like into Y2HGold strain (huaaobio). The mated strains were grown on double-selection medium (DDO) without leucine or tryptophan (SD/-Leu/-Trp) at 30°C for 3 days. A two-hybrid survival test for transformed yeast cells was conducted on selective quadruple dropout medium (QDO) (SD/adenine [-Ade]/histidine [-His]/-Leu/-Trp) supplied with 5-Bromo-4-chloro-3-indolyl *α* -D-galactopyranoside at 30°C for 3 days.

### Glutathione-S-transferase (GST) pull-down

In the GST pull-down assays, three fused proteins—pGEX-4T-1-PGRP-LC (GST-PGRP-LC), pGEX-4T-1-PGRP-LE (GST-PGRP-LE), and pGEX-4T-1-IMD (GST-IMD)—were used as bait proteins. The pET28a-Pirk-like fused protein (His-Pirk-like) was used as a prey protein, and empty vectors used as controls. Glutathione Sepharose 4B (GE Healthcare) beads were washed six times with 1× PBS to release the alcohol; then, 1 ml supernatant of bacteria containing GST, GST-PGRP-LC, GST-PGRP-LE, or GST-IMD was added. The supernatant was removed after incubating at 4°C for 2 h, and the beads were washed six times with 1× PBS (with 1% Triton X-100). His-pET28a or His-Pirk-like containing lysate was incubated with washed beads at 4°C for 4 h and washed six times with 1× PBS (with 1% Triton X-100) to remove unbound proteins. Subsequently, the proteins immobilized on the beads were tested using western blots. The following antibodies were used: anti-His (1:5000), anti-GST (1:3000), and anti-rabbit IgG (H & L)-HRP conjugated (1:10000).

### Western blot analysis

Fat bodies, ovaries, and cells were subjected to the indicated treatments and lysed with RIPA lysis Buffer (CWBIO). All lysates were electrophoresed by means of SDS-polyacrylamide gel electrophoresis (SDS-PAGE), then proteins were transferred to a PVDF membrane (voltage: 70 V; time: 90 min). The membranes were incubated in Starting Block T20 (PBS) Blocking Buffer (Pierce) with primary antibody for 2 h at room temperature (RT) and subjected to four 10-min washes with Tris-buffered saline Tween. The washed membranes were immersed in secondary antibody for 2 h at RT and washed again. SuperSignal Pico plus (Thermo Fisher Science) was used to detect protein expression. Proteins were immunoblotted with the following antibodies: anti-GAPDH (1:2500), anti-beta-Actin-HRP (1:2500), anti-V5, anti-His, anti-GST (1:2500), anti-Rel2 (1:4000), anti-PGRP-LC (1:4000), anti-Vg (1:4000), anti-mouse IgG(H&L)-HRP Conjugated (1:10000) and anti-rabbit IgG(H&L)-HRP Conjugated (1:10000).

To analyze nuclear translocation of Rel2, the cytosolic and nuclear proteins were extracted using Nuclear and Cytoplasmic Extraction Reagents (Thermo Fisher Science), following manufacturer’s instructions. The lysates were electrophoresed and immunoblotted with the following antibodies: anti-Rel2 (1:4000) and anti-Histone H3 (1:10000). The antibodies used are listed in the S1 Table.

### Chromatin immunoprecipitation and quantitative PCR (ChIP-qPCR)

Aag2 cells were co-transfected with pAc5.1b-EcR and -USP vectors for 40 h, then treated with 20E for 8 h. Subsequently, the cells were treated with 1% paraformaldehyde (PFA) for 10 min at RT and harvested to isolate and lyse nuclear fractions, as previously described [55]. Fragmented chromatin was obtained using Bioruptor plus (Diagenode) and immunoprecipitated overnight at 4°C with 3 μg anti-V5, and IgG antibodies. Purified ChIP or input DNA was quantified using Qubit 2.0 then amplified by means of qRT-PCR.

### Amyloid aggregate staining

Aag2 cells were transfected with pAc5.1b-PGRP-LC, -PGRP-LE, -IMD, or pAc5.1b no-load vectors for 48 h. Then, the transfected cells were treated with 4% PFA in PBS for 30 min. Fixed cells were rinsed with 1× PBS three times and permeabilized with 0.5%-Triton-X-100 for 10 min. Then, the cells were gently washed with 1× PBS, blocked at RT for 1 h with Blocking solution (3% BSA) (Invitrogen), and incubated overnight at 4°C with anti-V5 (1:700) or anti-HA (1:700) antibody. After washing several times with 1× PBS and staining with anti-mouse Alexa Fluor 546 (Invitrogen) antibodies at RT for 2 h, the cells were washed again and stained with 30 mM ThT (Solarbio) in 3:7 ethanol:PBS for 40 min. Then, the cells were washed five times in PBS and mounted with ProLong Gold Antifade Reagent (Invitrogen). The cells were imaged using a Zeiss LSM710 20× immersion objective using sequential scanning. ThT was excited at 488 nm and emission was detected at 493–556 nm. HA and V5 were excited at 561 nm and emission detected at 566–680 nm.

### RNA-Seq analyses of bacterial-infected iEcR mosquitoes

EcR interference and infection were carried out as described above, and then fat bodies were dissected at 12 h post infection. Total RNA was extracted using Trizol reagent, following a method described previously [50]. All RNA samples had an RNA Integrity Number (RIN) > 7.0, 28S/18S ≥ 1; OD260/OD280 = 1.8–2.0. RNA-Seq libraries and sequencing were generated by Wuhan Igenebook Biotechnology (Wuhan, China). The clean reads were mapped to the *Ae*. *aegypti* genome database (https://vectorbase.org/vectorbase/app/downloads/release-49/AaegyptiLVP_AGWG). The number of perfect clean reads corresponding to each gene was calculated and normalized to fragments per kilobase per million mapped reads (FPKM). Based on the expression levels, differentially expressed IMRGs were identified using false discovery rate (FDR) < 0.05 and log_2_(fold change) > 1 or log_2_(fold change) < -1. Heap maps (S2 Table) were constructed using the R package. UpSet diagrams and volcano plots were generated for expression level analysis of the IMRGs.

### RNA-Seq analyses of infected WT and Pirk-like knockout mosquitoes

At 12 h after a blood meal, WT and Pirk-like^-/-^ mosquitoes were infected with *E*. *cloacae* or PBS. The fat bodies were dissected at 12 h and 24 h post infection. RNA-Seq data were generated and processed as described above. We analyzed the average expression levels of genes involved in FoxO and the oocyte meiosis pathway in Pirk-like^-/-^_PBS 12 h versus WT_PBS 12 h mosquitoes using the GSEA method. Heat maps (S3 Table), UpSet diagram, and volcano plots were generated for expression level analysis of the IMRGs. All raw data of RNA-Seq files are available from the NCBI SRA database (PRJNA817039, PRJNA817040) and Science Data Bank (31253.11.sciencedb.j00139.00010 and 31253.11.sciencedb.j00139.00011).

### Immunohistochemistry and quantification of fluorescence signal intensity

Fat bodies were dissected from the control and treated female mosquitoes and fixed in 4% PFA for 1 h at RT. Tissue samples were washed several times in 1× PBS and blocked in 1× PBS containing 0.5% normal goat serum for 1 h at RT. Tissues were then stained with primary antibody overnight at 4°C and washed and incubated with secondary antibody for 2 h at RT. After washing and staining for 10 min with Hochest33258 (0.2 μg/mL in PBS), the tissues were washed again and mounted on an adhesion microscope slide (Citotest) with ProLong Gold Antifade Reagent (Invitrogen). The following antibodies were used: anti-PGRP-LC (1:700), anti-Rel2 (1:700), anti-Vg (1:700), anti-mouse Alexa Fluor 546 (1:1000), anti-rabbit Alexa Fluor 488 (1:1000), and anti-mouse Alexa Fluor 568 (1:1000). The draw spline contour tool of ZEN software was used to mark the cytoplasmic or nuclear regions to obtain the average signal intensity per pixel from confocal images. These values were used to quantify PGRP-LC, Rel2 or Vg expression in the cytoplasm and Rel2 expression in the nuclei.

### Embryonic injection and mutant line generation

The sgRNAs of *Pirk-like* were designed using the Zhang Lab (USA) website (https://zlab.bio/guide-design-resources) and synthesized using the T7 RiboMAX Express RNAi system (Promega). We purified sgRNAs with the MEGAclear Transcription Clean-Up Kit (Invitrogen) following the manufacturer’s protocol. After the cleavage activity of Cas9 protein (PNA Bio) was verified *in vitro*, we chose the appropriate sgRNAs (50 ng/μL), mixed these with Cas9 protein (333 ng/μL) at 37°C for 20 min, and then microinjected the mixture into the posterior pole of embryos using an Eppendorf FemtoJet 4i. The injected embryos hatched after 5 days and were reared to adulthood under standard conditions according to the described protocols. G0 mosaic females were crossed with WT males to produce the G1 generation. The G1 heterozygotes identified by sequencing were hybridized with WT to generate the G2 generation. The heritable genotype was confirmed by TA cloning. The heterozygotes with the same genotype in G2 were self-crossed, and Pirk-like knockout strains were obtained by homozygous self-hybridization for at least three generations.

## Supporting information

S1 Fig Rel2 expression is regulated by ecdysone signaling in the fat body. (A) Silence efficiency of EcR was measured using qRT-PCR. Data are shown as mean ± SEM. ****p < 0.0001; one-way ANOVA, followed by Bartlett’s test. (B and C) qRT-PCR analysis of *PGRP-LC* (B) and *Rel2* (C) in iEcR and iEGFP mosquitoes infected by *E*. *cloacae* for 12 h. Bar plots are shown as mean ± SEM. ****p < 0.0001 (one-way ANOVA followed by Bartlett’s test). Data are from three biological replicates. (D and E) Confocal images (D) and corresponding quantification of Rel2 (E) expression in iEcR and iEGFP female fat bodies infected with *E*. *cloacae* for 24 h after blood meal. Statistical analysis was performed using t-test. ***p < 0.0001.
(TIF)

S2 Fig Ecdysone suppresses AMP gene expression. (A-C) Volcano plots of differentially expressed IMRGs in iEGFP and iEcR mosquito fat bodies after *E*. *cloacae* infection. Each gene is marked as a dot; the broken lines indicate the marginal lines separating differentially expressed IMRGs from non-differentially expressed IMRGs, with the horizontal broken lines denoting the p value threshold (p < 0.05) and the vertical broken lines representing the fold change cutoff (log_2_(fold change) > 1 or log_2_(fold change) < -1). (D) Blood-fed iEGFP or iEcR mosquitoes were stimulated for 12 h with OD600 = 1 *E*. *cloacae* or PBS and then analyzed using qRT-PCR for *ATT*, *Dpt*, *CecB*, *DefC* and *GAM* transcriptions. Data are shown as mean ± SEM. ****p < 0.0001; one-way ANOVA followed by Bartlett’s test. Data are from at least three biological replicates.
(TIF)

S3 Fig Effects of 20E on IMD pathway genes, related to Fig 3. (A) Pairwise sequence alignment of *Ae*. *aegypti* Pirk-like and *D*. *melanogaster* Pirk. (B) Phylogenetic tree analysis of the protein sequence of *Ae*. *aegypti* Pirk-like with Pirk from other insects. The maximum likelihood method was used for comparison, and the scale bar represents genetic distance.
(TIF)

S4 Fig 20E, EcR and USP bind to the EcRE1 of Pirk-like. EMSA shows the binding of EcR to the biotin-labeled EcRE1 probe. Nuclear protein extracts from Aag2 cells co-expressing EcR and USP. Competitor (unlabeled 32-bp probe) and anti-EcR antibody were added as indicated.
(TIF)

S5 Fig Conserved cRHIM motifs in the *Ae*. *aegypti* IMD pathway. (A) Multiple sequence alignment of cRHIMs from *D*. *melanogaster*, *Ae*. *aegypti* and human RHIM. The four core amino acids are boxed with red. The highly conserved amino acids are shown in different colors. (B-D) Yeast two-hybrid assays, pGADT7-PGRP-LC, pGADT7-PGRP-LE, and pGADT7-IMD were used as baits and pGBKT7-Pirk-like was used as prey. Yeast was grown on DDO and QDO media at 30°C for 3–5 days. pGADT7-T and pGBKT7-53 or pGBKT7-Lam co-transform strain were used as negative and positive controls, respectively (B); auto-activation and interaction analysis of Pirk-like (C); PGRP-LC and IMD were auto-activated (D). (E) Effect of EcR silencing on amyloid formation in mosquito fat body infected as indicated after blood meal. Scale bar: 10 μm.
(TIF)

S6 Fig Pirk-like is a feedback inhibitor of IMD pathway. (A) Density plot showing the effect of Pirk-like knockout on IMRG expression after *E*. *cloacae* or PBS treatment. Gray shaded area indicates values used to define differentially expressed genes or resistant genes. (B) Venn diagram showing the core genes enriched among the differentially expressed IMRG shared by different treatment groups. (C-E) Volcano plots showing IMRG expression of treatment groups compared with control (WT_PBS 12 h). Each gene is marked as a dot; the red plots represent significantly enriched genes. The blue plots represent downregulated genes. The broken lines indicate the marginal lines selecting DEGs, with the horizontal broken lines denoting the p value threshold (p < 0.001) and the vertical broken lines representing the fold change cutoff in log2 scale. (F) qRT-PCR was used to determine the transcripts of *Pirk-like* in iEGFP and iRel2 mosquitoes either uninfected or infected with *E*. *cloacae* for 12 h. Data are shown as mean ± SEM. ****p < 0.0001; one-way ANOVA, followed by Bartlett’s test. Data represent at least three independent experiments. (G and H) UpSet plots showing the interactions between different KEGG pathways. Selected upregulated (G) and downregulated (H) KEGG pathways. (I) WT or Pirk-like^-/-^ mosquitoes were stimulated for 12 h with OD600 = 1 *E*. *cloacae* or PBS at PE stage and then analyzed using qRT-PCR for *CecB*, *CecG*, *DefC*, *DefD* and *GAM* transcriptions. Data are shown as mean ± SEM. ****p < 0.0001; one-way ANOVA followed by Bartlett’s test. Data are from at least three biological replicates.
(TIF)

S7 Fig Pirk-like knockdown decreased egg production. (A-B) GSEA enrichment plots for the FoxO signaling pathway (A) and oocyte meiosis pathways (B) in Pirk-like^-/-^_PBS 12 h compared with control (WT_PBS 12 h). Statistical analysis: Normalized Enrichment Score (NES) and p value. (C) Ovary development of WT and Pirk-like^-/-^ mosquitoes at 72 h PE. Data represent at least three biological replicates. (D) qRT-PCR analysis of *Vg* in WT, Pirk-like^+/-^, and Pirk-like^-/-^ mosquito fat bodies. Bar plots represent mean ± SEM. Data are from three biological replicates. ****p < 0.0001; one-way ANOVA, followed by Bartlett’s test. (E) WT, Pirk-like^+/-^, and Pirk-like^-/-^ mosquito ovaries were analyzed using western blots for Vg and GAPDH (control) at 36 h PBM. Data represent at least three independent experiments. (F) Comparison of the egg deposition between iEGFP and iPirk-like mosquitoes. Data represent three biological replicates and are shown as mean ± SEM. (G) Comparison of the egg deposition among iEGFP_PBS, iEGFP_Ec, iEcR_PBS, and iEcR_Ec mosquitoes. Data represent three biological replicates and are shown as mean ± SEM. (H) Fat bodies of iEGFP and iEcR mosquitoes either uninfected or infected with *E*. *cloacae* (OD600 = 0.8) for 12 h were analyzed by means of western blots with Vg antibody.
(TIF)

S1 Table Used antibodies and oligonucleotides. (PDF)

S2 Table Differentially expressed IMRGs in iEcR and iEGFP mosquito fat bodies with *E*. *cloacae* infection. (PDF)

S3 Table Differentially expressed IMRGs in Pirk-like^-/-^ and WT mosquito fat bodies with *E*. *cloacae* infection. (PDF)
