# Supplementary figures and images for: Ecdysone signaling mediates the trade-off between immunity and reproduction via suppression of amyloids in the mosquito Aedes aegypti
Source: PLoS Pathog. 2022 Sep 22;18(9):e1010837. doi: 10.1371/journal.ppat.1010837 (PMC9531809; doi:10.1371/journal.ppat.1010837)

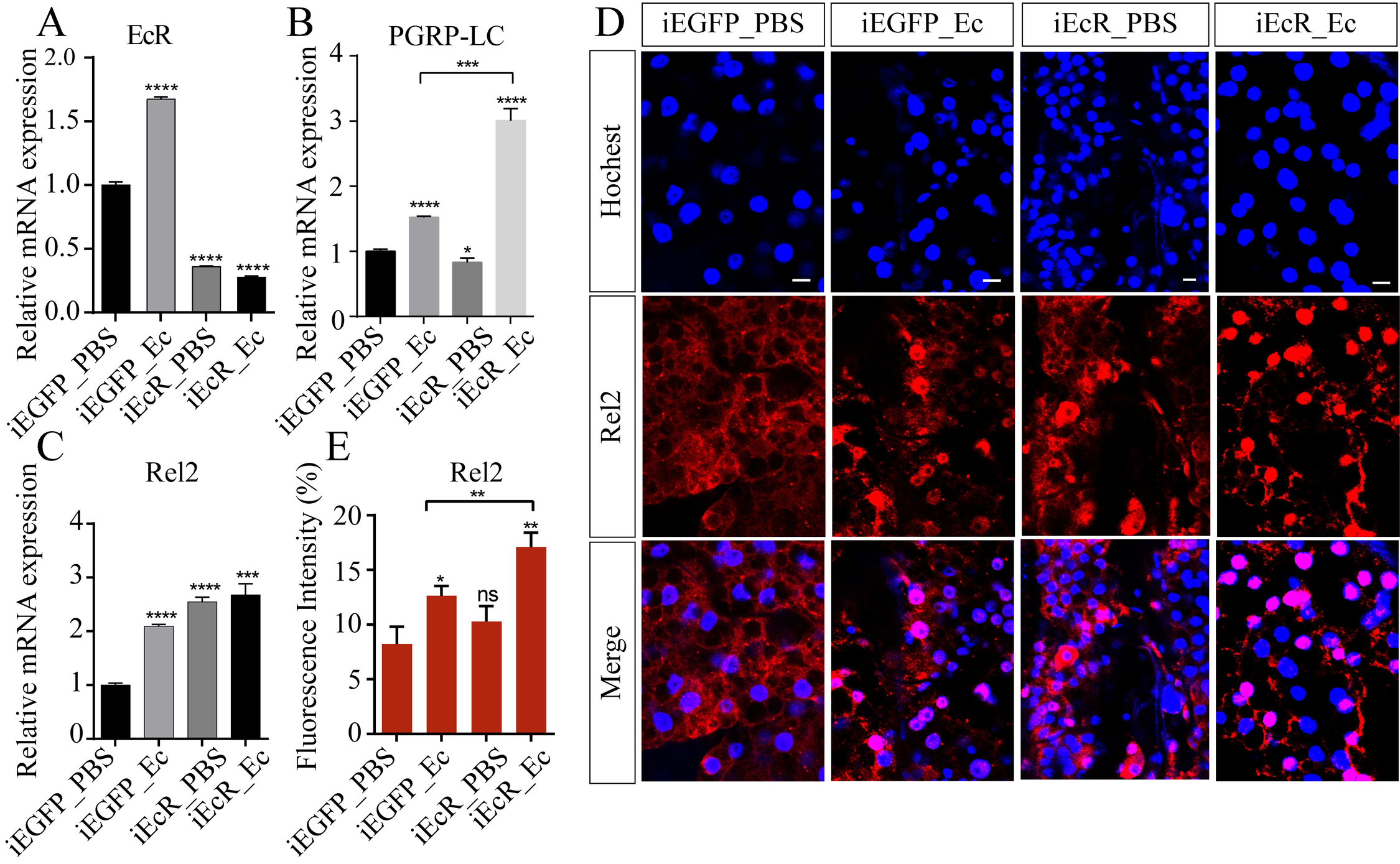

Supplement: S1 Fig — (A) Silence efficiency of EcR was measured using qRT-PCR. Data are shown as mean ± SEM. ****p < 0.0001; one-way ANOVA, followed by Bartlett’s test. (B and C) qRT-PCR analysis of PGRP-LC (B) and Rel2 (C) in iEcR and iEGFP mosquitoes infected by E. cloacae for 12 h. Bar plots are shown as mean ± SEM. ****p < 0.0001 (one-way ANOVA followed by Bartlett’s test). Data are from three biological replicates. (D and E) Confocal images (D) and corresponding quantification of Rel2 (E) expression in iEcR and iEGFP female fat bodies infected with E. cloacae for 24 h after blood meal. Statistical analysis was performed using t-test. ***p < 0.0001. (TIF) [file ppat.1010837.s001.tif]

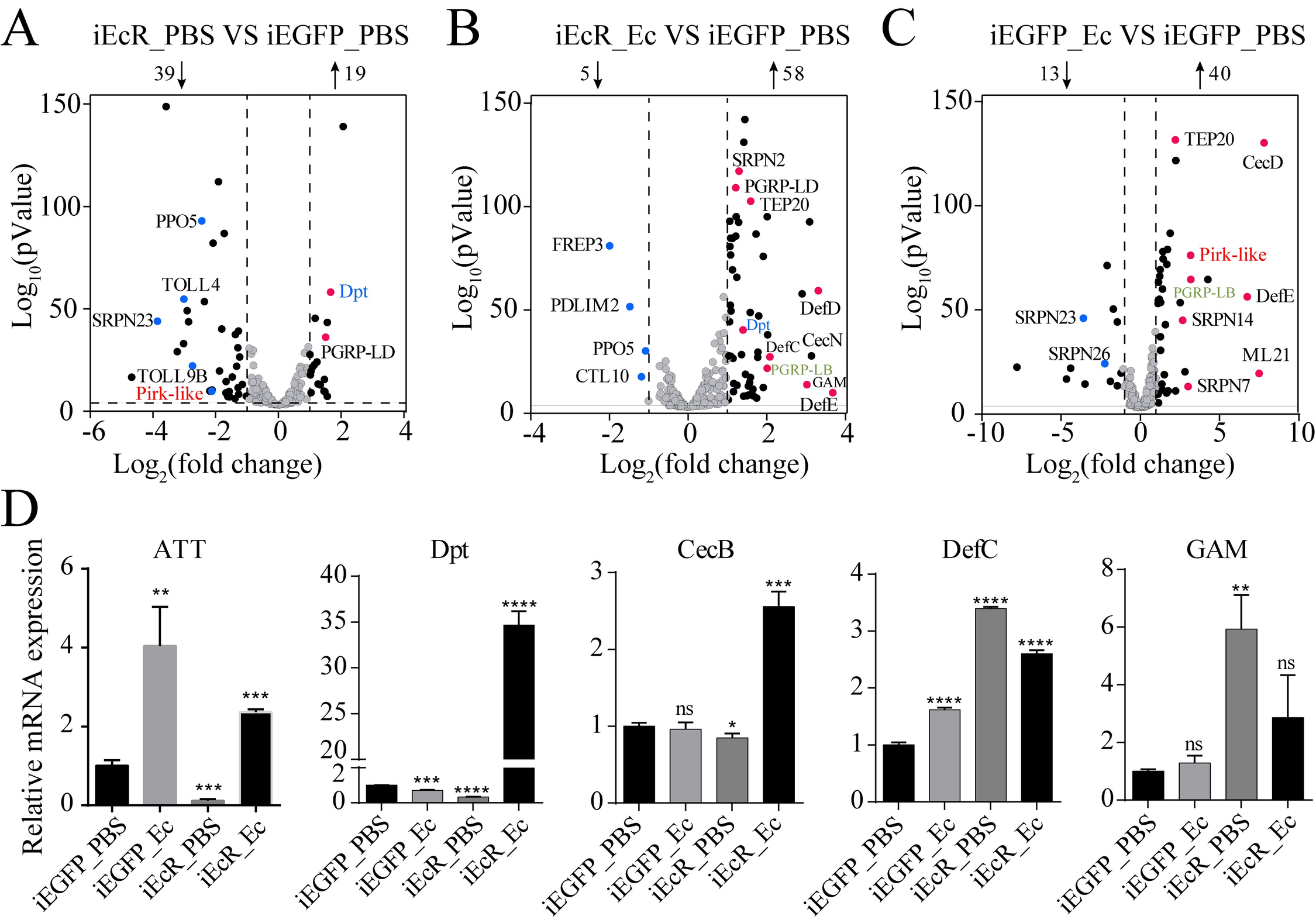

Supplement: S2 Fig — (A-C) Volcano plots of differentially expressed IMRGs in iEGFP and iEcR mosquito fat bodies after E. cloacae infection. Each gene is marked as a dot; the broken lines indicate the marginal lines separating differentially expressed IMRGs from non-differentially expressed IMRGs, with the horizontal broken lines denoting the p value threshold (p < 0.05) and the vertical broken lines representing the fold change cutoff (log2(fold change) > 1 or log2(fold change) < -1). (D) Blood-fed iEGFP or iEcR mosquitoes were stimulated for 12 h with OD600 = 1 E. cloacae or PBS and then analyzed using qRT-PCR for ATT, Dpt, CecB, DefC and GAM transcriptions. Data are shown as mean ± SEM. ****p < 0.0001; one-way ANOVA followed by Bartlett’s test. Data are from at least three biological replicates. (TIF) [file ppat.1010837.s002.tif]

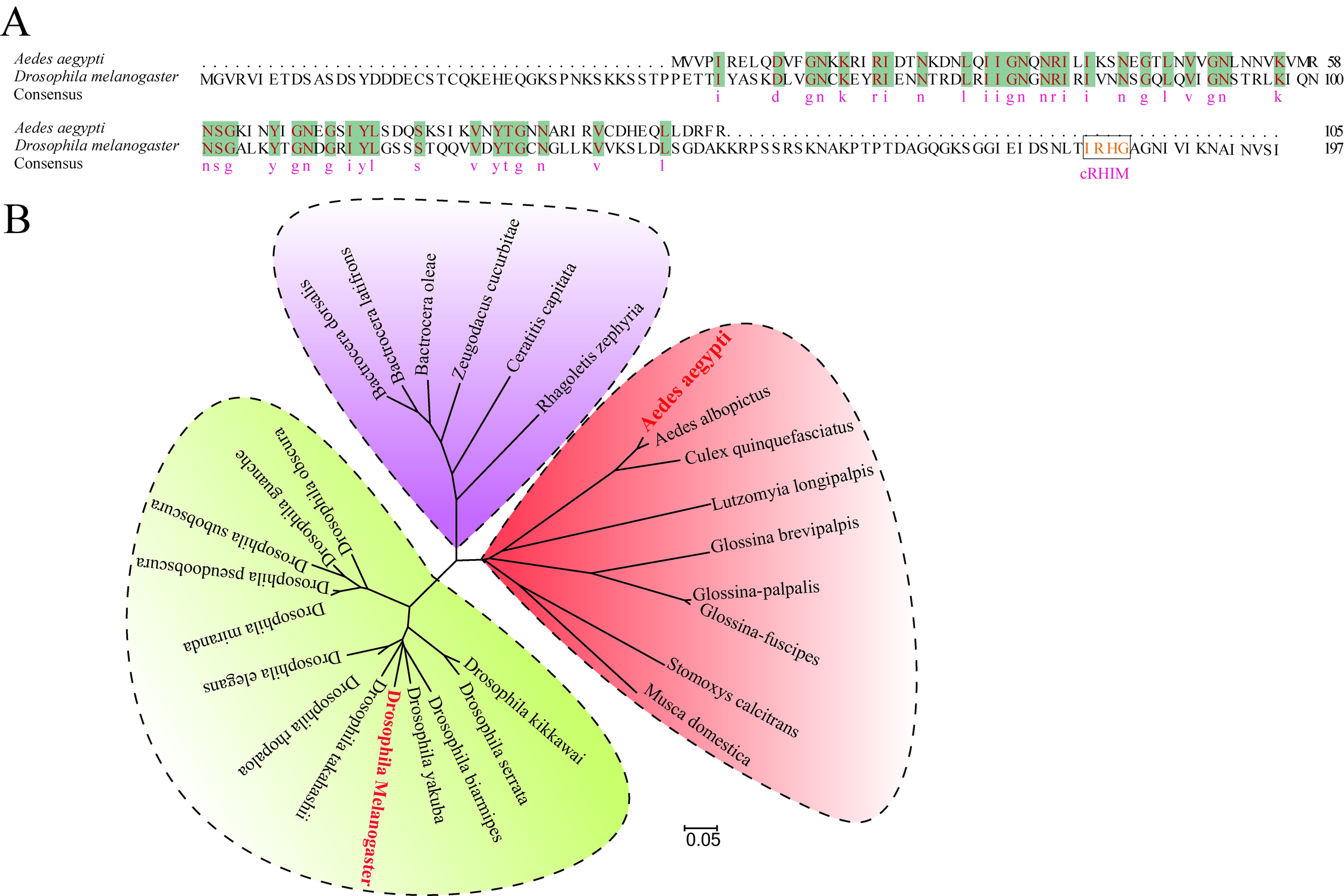

Supplement: S3 Fig — (A) Pairwise sequence alignment of Ae. aegypti Pirk-like and D. melanogaster Pirk. (B) Phylogenetic tree analysis of the protein sequence of Ae. aegypti Pirk-like with Pirk from other insects. The maximum likelihood method was used for comparison, and the scale bar represents genetic distance. (TIF) [file ppat.1010837.s003.tif]

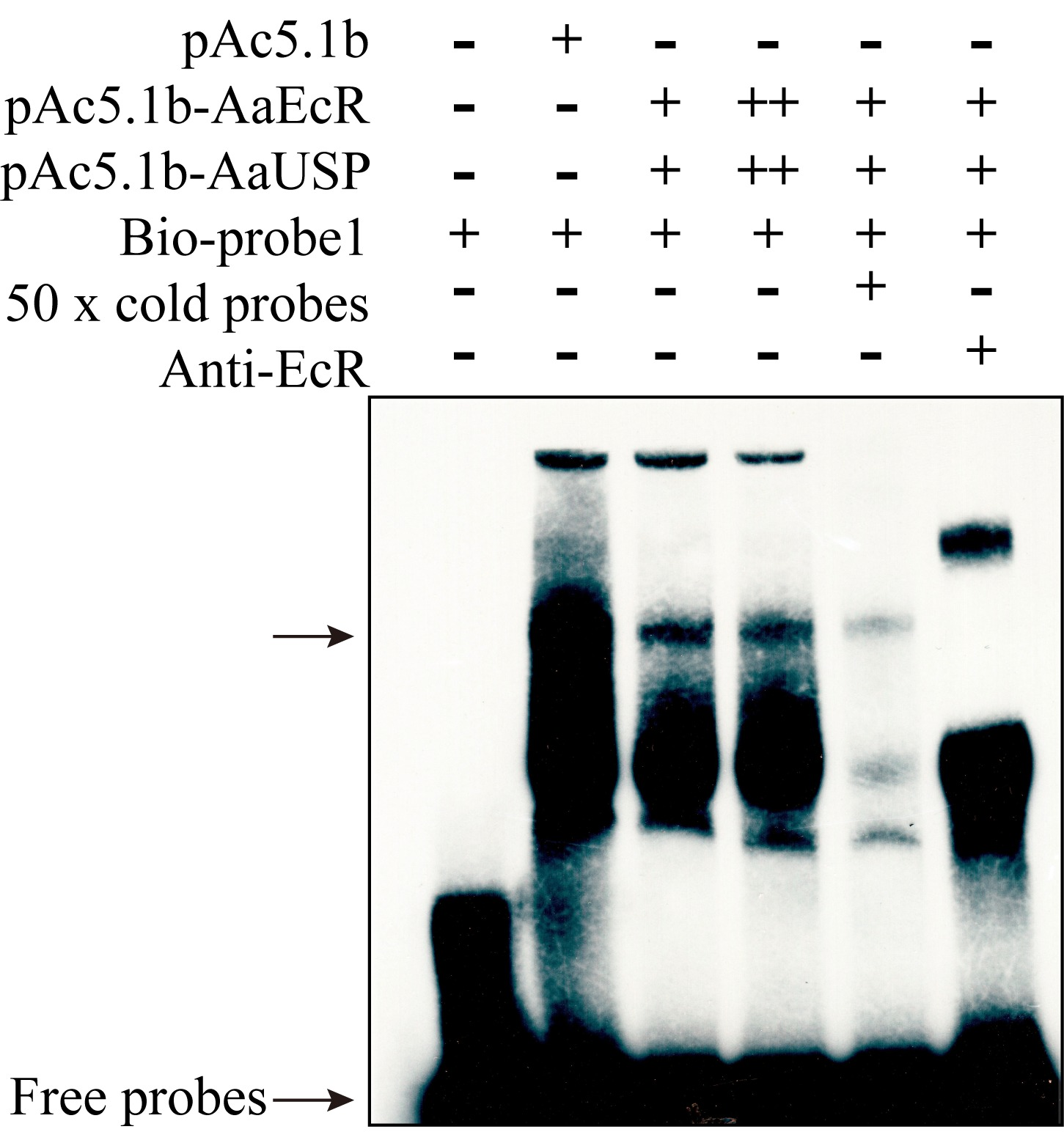

Supplement: S4 Fig — EMSA shows the binding of EcR to the biotin-labeled EcRE1 probe. Nuclear protein extracts from Aag2 cells co-expressing EcR and USP. Competitor (unlabeled 32-bp probe) and anti-EcR antibody were added as indicated. (TIF) [file ppat.1010837.s004.tif]

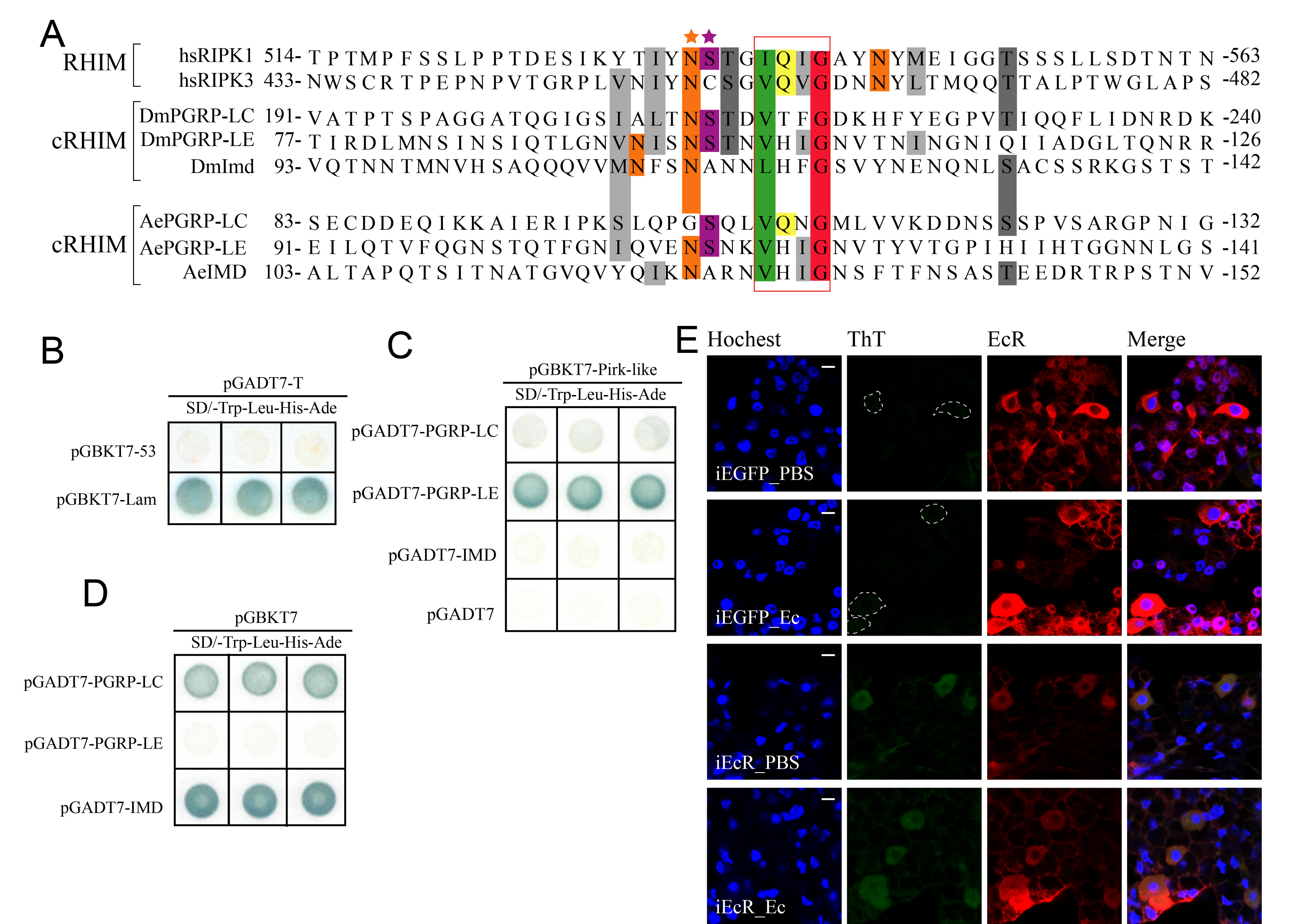

Supplement: S5 Fig — (A) Multiple sequence alignment of cRHIMs from D. melanogaster, Ae. aegypti and human RHIM. The four core amino acids are boxed with red. The highly conserved amino acids are shown in different colors. (B-D) Yeast two-hybrid assays, pGADT7-PGRP-LC, pGADT7-PGRP-LE, and pGADT7-IMD were used as baits and pGBKT7-Pirk-like was used as prey. Yeast was grown on DDO and QDO media at 30°C for 3–5 days. pGADT7-T and pGBKT7-53 or pGBKT7-Lam co-transform strain were used as negative and positive controls, respectively (B); auto-activation and interaction analysis of Pirk-like (C); PGRP-LC and IMD were auto-activated (D). (E) Effect of EcR silencing on amyloid formation in mosquito fat body infected as indicated after blood meal. Scale bar: 10 μm. (TIF) [file ppat.1010837.s005.tif]

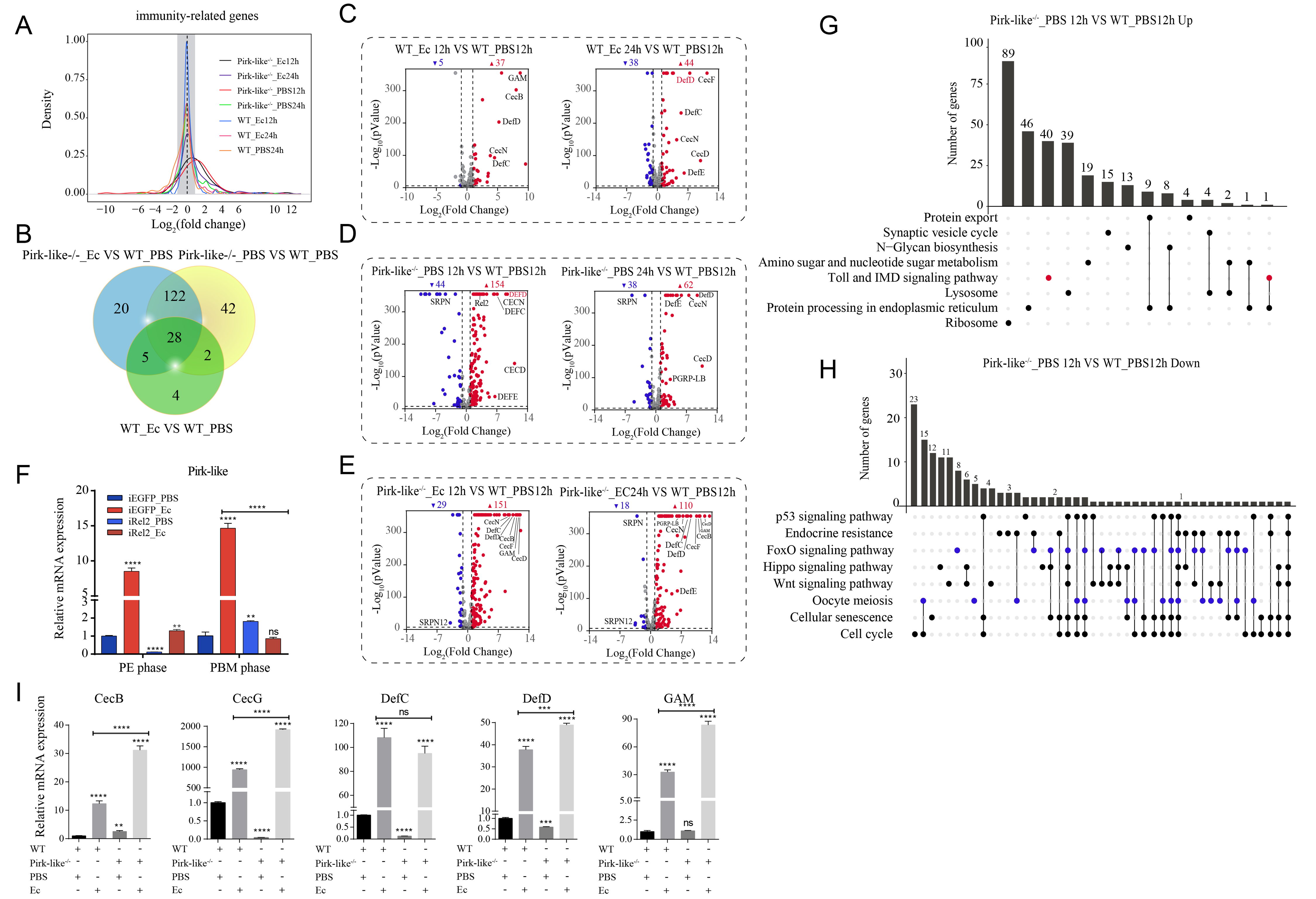

Supplement: S6 Fig — (A) Density plot showing the effect of Pirk-like knockout on IMRG expression after E. cloacae or PBS treatment. Gray shaded area indicates values used to define differentially expressed genes or resistant genes. (B) Venn diagram showing the core genes enriched among the differentially expressed IMRG shared by different treatment groups. (C-E) Volcano plots showing IMRG expression of treatment groups compared with control (WT_PBS 12 h). Each gene is marked as a dot; the red plots represent significantly enriched genes. The blue plots represent downregulated genes. The broken lines indicate the marginal lines selecting DEGs, with the horizontal broken lines denoting the p value threshold (p < 0.001) and the vertical broken lines representing the fold change cutoff in log2 scale. (F) qRT-PCR was used to determine the transcripts of Pirk-like in iEGFP and iRel2 mosquitoes either uninfected or infected with E. cloacae for 12 h. Data are shown as mean ± SEM. ****p < 0.0001; one-way ANOVA, followed by Bartlett’s test. Data represent at least three independent experiments. (G and H) UpSet plots showing the interactions between different KEGG pathways. Selected upregulated (G) and downregulated (H) KEGG pathways. (I) WT or Pirk-like-/- mosquitoes were stimulated for 12 h with OD600 = 1 E. cloacae or PBS at PE stage and then analyzed using qRT-PCR for CecB, CecG, DefC, DefD and GAM transcriptions. Data are shown as mean ± SEM. ****p < 0.0001; one-way ANOVA followed by Bartlett’s test. Data are from at least three biological replicates. (TIF) [file ppat.1010837.s006.tif]

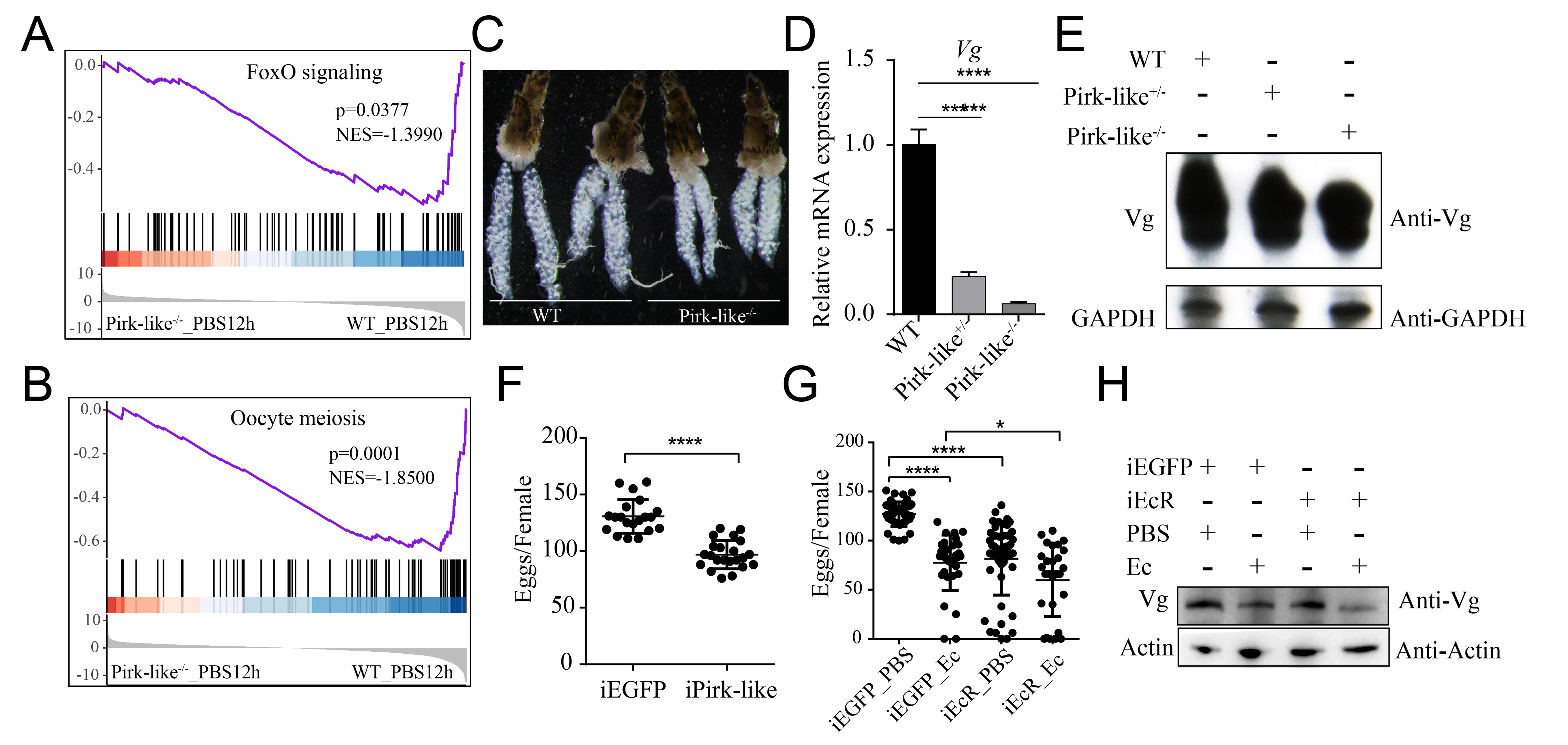

Supplement: S7 Fig — (A-B) GSEA enrichment plots for the FoxO signaling pathway (A) and oocyte meiosis pathways (B) in Pirk-like-/-_PBS 12 h compared with control (WT_PBS 12 h). Statistical analysis: Normalized Enrichment Score (NES) and p value. (C) Ovary development of WT and Pirk-like-/- mosquitoes at 72 h PE. Data represent at least three biological replicates. (D) qRT-PCR analysis of Vg in WT, Pirk-like+/-, and Pirk-like-/- mosquito fat bodies. Bar plots represent mean ± SEM. Data are from three biological replicates. ****p < 0.0001; one-way ANOVA, followed by Bartlett’s test. (E) WT, Pirk-like+/-, and Pirk-like-/- mosquito ovaries were analyzed using western blots for Vg and GAPDH (control) at 36 h PBM. Data represent at least three independent experiments. (F) Comparison of the egg deposition between iEGFP and iPirk-like mosquitoes. Data represent three biological replicates and are shown as mean ± SEM. (G) Comparison of the egg deposition among iEGFP_PBS, iEGFP_Ec, iEcR_PBS, and iEcR_Ec mosquitoes. Data represent three biological replicates and are shown as mean ± SEM. (H) Fat bodies of iEGFP and iEcR mosquitoes either uninfected or infected with E. cloacae (OD600 = 0.8) for 12 h were analyzed by means of western blots with Vg antibody. (TIF) [file ppat.1010837.s007.tif]
