# Supplementary material for: Ecdysone signaling mediates the trade-off between immunity and reproduction via suppression of amyloids in the mosquito Aedes aegypti
Source: PLoS Pathog. 2022 Sep 22;18(9):e1010837. doi: 10.1371/journal.ppat.1010837 (PMC9531809; doi:10.1371/journal.ppat.1010837)
Supplement: S1 Table — (PDF) [file ppat.1010837.s008.pdf]

S1Table. Used antibodies and oligonucleotides

Antibodies or Oligonucleotides	SOURCE	IDENTIFIER
Antibodies		
Anti-V5	Thermo Fisher Science	Cat# R96025
Anti-GST	Proteintech	Cat# 10000-0-AP-100
Anti-His	TIANGEN	Lot# U9023
Anti-Vg	Beijing Protein Innovation	N/A
Anti-PGRP-LC	Beijing Protein Innovation	N/A
Anti-Rel2	Beijing Protein Innovation	N/A
Anti-GAPDH	EASYBIO	Cat# BE0024-10
Anti-Histone H3	EASYBIO	Cat# BE3021-100
Anti-beta-Actin-HRP	EASYBIO	Cat# BE0033-100
Anti-rabbit lgG(H&L)-HRP Conjugated	EASYBIO	Cat# BE0101-100
Anti-mouse lgG(H&L)-HRP Conjugated	EASYBIO	Cat# BE0102-100
Anti-HA	EASYBIO	Cat# BE2007-100
Anti-mouse Alexa Fluor 546	Invitrogen	Cat# A11030
Anti-rabbit Alexa Fluor 488	Invitrogen	Cat# A27034
Anti-rabbit Alexa Fluor 568	Invitrogen	Cat# A11036
Oligonucleotides		
EcRE1 of Pirk-like: AATTTCATATAACTCAAAAAGTAAATAACTTG	This paper	N/A
EcRE2 of Pirk-like: TCTTTTGAGTTTGGTGAATTCAAGAA	This paper	N/A

EcRE2 mutant of Pirk-like: TCTTTTGAGTCTGGCAAATTCAAGAA	This paper	N/A
Primer *EcR* forward: GGTTATGATGTTGCGAATGG	This paper	N/A
Primer *EcR* reverse: GCAGGTCCTCTATCGTGTCC	This paper	N/A
Primer *Dpt* forward: TGTCCATCCGAGTGAGACGT	This paper	N/A
Primer *Dpt* reverse: CTCCCTGAAATCCACCAAAA	This paper	N/A
Primer *CecB* forward: AAGCTGGTCGGCTGAAGAAG	This paper	N/A
Primer *CecB* reverse: ATCTTCCCAGTCCCTTGATG	This paper	N/A
Primer *DefC* forward: GCCTCAGTGCAATCTTCACA	This paper	N/A
Primer *DefC* reverse: CGTTTCAAGCGGAAGTTTTC	This paper	N/A
Primer *GAM* forward: GCCAAAACCTGTTCCTCTTG	This paper	N/A
Primer *GAM* reverse: CGATGTAGCATTCGGTGATG	This paper	N/A
Primer *Pirk-like* forward: TAAATCACCGCTCGCTACAT	This paper	N/A
Primer *Pirk-like* reverse: TGCAACTGACTATCGAACCC	This paper	N/A
Primer *PGRP-LC* forward: AACTTTCTGATCGGAGGTGA	This paper	N/A
Primer *PGRP-LC* reverse: ACGCTATGCCAATACTGTCG	This paper	N/A
Primer *IMD* forward: GCAACAGGTGTCCAGGTTTA	This paper	N/A

Primer *IMD* reverse: ACTCCATCATCTGCCGAATT	This paper	N/A
Primer *Rel2* forward: GGATGCGGTGTCGGTTGGTC	This paper	N/A
Primer *Rel2* reverse: CGTCCTTGGCTTGGCTGTTG	This paper	N/A
Primer *Vg* forward: CCAGAAGACGTGAGCATTCA	This paper	N/A
Primer *Vg* reverse: TGGCGCAGATGATAGAACAG	This paper	N/A
Primer *EcRA* forward: GCACAGGGACAGCAGGTGAT	This paper	N/A
Primer *EcRA* reverse: AATCGTGGTAGCATTGAGGG	This paper	N/A
Primer *EcRB* forward: GTGGGAGTGTTGTTGAGTTT	This paper	N/A
Primer *EcRB* reverse: CACCATTGTTGGACCATCTT	This paper	N/A
