# Supplementary material for: Ecdysone signaling mediates the trade-off between immunity and reproduction via suppression of amyloids in the mosquito Aedes aegypti
Source: PLoS Pathog. 2022 Sep 22;18(9):e1010837. doi: 10.1371/journal.ppat.1010837 (PMC9531809; doi:10.1371/journal.ppat.1010837)
Supplement: S2 Table — (PDF) [file ppat.1010837.s009.pdf]

S2Table. Differentially expressed IMRGs in iEcR and iEGFP mosquito fat bodies with *E. cloacae* infection. Genes with a minimum fold change of 2-fold and a false discovery rate-adjusted p-value (q-value) of < 0.05 were considered differentially expressed.

GeneID	iEcR_Ec	iEcR_PBS	iEGFP_Ec	iEGFP_PBS	GeneName
AAEL002301	106.363	92.39	94.6767	43.8167	CLIPA5
AAEL000074	99.4367	74.7267	74.3867	37.1267	CLIPB1
AAEL014349	30.3233	28.2467	24.6067	12.43	CLIPB15
AAEL001084	1.93667	0.913333	1.42	0.706667	CLIPB21
AAEL014140	43.3233	37.17	22.66	17.94	CLIPB24
AAEL006674	179.89	118.733	67.2067	75.21	CLIPB29
AAEL000099	78.4867	57.9033	49.1833	31.3333	CLIPB33
AAEL004948	6.76667	2.46667	2.11	2.65667	CLIPC14
AAEL000224	2.69	1.37	1.63	0.853333	CLIPD7
AAEL011453	18.2867	9.40333	14.9	7.90667	CTL14
AAEL013853	1.4	0.47	0.396667	0.406667	CTLGA2
AAEL014357	0.78	0.393333	0.313333	0.306667	CTLSE2
AAEL004522	2.45	0.103333	0.206667	0.253333	GAM
AAEL012069	94.75	66.14	67.4133	37.78	GPXH1
AAEL011764	0.693333	0.14	0.21	0.223333	PPO10
AAEL013496	3.07	0.533333	0.693333	0.79	PPO8
AAEL002720	98.5533	74.6233	60.7233	31.5933	SRPN20
AAEL013936	27.8633	17.8367	21.0867	11.5067	SRPN4A
AAEL013441	3.70333	1.23333	1.06667	1.25	TOLL9A
AAEL011009	1.26	0.57	0.46	0.383333	FREP8
AAEL011007	28.63	7.84	13.5733	6.18667	FREP9
AAEL014078	109.483	61.15	96.68	38.9733	SRPN2
AAEL002585	50.5067	38.31	35.6233	13.34	CLIPA11
AAEL002629	223.743	142.97	69.0533	38.0667	CLIPA6
AAEL008668	4.61333	2.59333	3.18	0.993333	CLIPB22
AAEL000037	78.8967	84.6267	46.8033	15.8733	CLIPB35
AAEL022578	252.703	222.843	247.523	82.4433	CLIPB8
AAEL011991	6.73	5.22333	4.87333	1.71	CLIPC1
AAEL009338	3.10667	3.54667	3.44667	6.18667	CTL10
AAEL005482	22.54	8.17667	14.78	2.31333	CTL18
AAEL018207	0.266667	0.133333	1.13333	0.466667	CTL8
AAEL011455	28.05	21.1367	7.11333	8.15333	CTLMA12
AAEL000563	6.46	8.09333	3.74333	2.51667	CTLMA15
AAEL003832	9.17333	0.746667	280.56	1.90333	DEFC
AAEL004833	28.86	38.4967	8.81	9.54667	DPT
AAEL006704	3.21	0.0233333	0.0966667	0.953333	FREP18
AAEL003294	7.34333	1.5	10.2733	25.3567	FREP3
AAEL003844	139.92	141.357	121.677	50.1533	GALE5
AAEL009906	4.17	3.03333	3.45	10.1067	PDLIM2

AAEL010171	2.36	1.76667	5.18667	0.51	PGRPLB
AAEL019745	158.48	215.73	89.54	59.6967	PGRPLD
AAEL021557	3.86333	0.33	11.3	1.11667	Pirk-like
AAEL013492	3	1.28333	4.15333	5.54	PPO5
AAEL002731	5.96333	2.44667	4.93	0.686667	SRPN14
AAEL011777	3.83667	6.19	8.61667	1.65333	SRPN8
AAEL002309	24.8833	3.83333	5.63	10.58	TPX4
AAEL006568	233.543	81.1833	54.4967	10.3733	SRPN
AAEL008118	0.46	0.22	0.406667	0.463333	cGAS
AAEL014137	5.61667	1.61667	6.00333	6.52667	CLIPB25
AAEL027429	0.546667	0.286667	0.463333	0.663333	CLIPB76
AAEL007992	3.17333	0.753333	2.77333	1.91	CLIPB78
AAEL007796	1.22	0.39	0.93	0.756667	CLIPD1
AAEL002124	5.87333	3.34333	8.97	6.17667	CLIPD6
AAEL018265	1.40667	0.82	1.47	1.55333	CTL9
AAEL019868	10.29	0.816667	8.19	4.9	FREP17
AAEL002354	0.256667	0.0866667	0.566667	0.31	HPX5
AAEL012380	2.38333	6.24	2.69	2.08333	PGRPLA
AAEL015116	1.70333	0.573333	0.996667	0.92	PPO1
AAEL011763	1.63667	0.466667	1.11667	1.19	PPO3
AAEL013501	2.45	0.47	2.09	2.74333	PPO4
AAEL020579	1.33667	0.343333	0.706667	0.796667	PPO9
AAEL027694	2.75	1.34	2.80667	3.95667	SCRASP3
AAEL005979	4.59	2.01333	4.97667	3.87667	SCRB3
AAEL000256	1.79667	0.966667	1.45	1.90667	SCRB9
AAEL009423	81.73	116.557	71.9133	44.6133	SCRBQ2
AAEL019958	1.48	0.263333	1.91333	1.96333	TEP21
AAEL015019	1.75	0.4	2.31333	2.51667	TOLL4
AAEL006649	3.68	2.58667	3.58667	5.25333	TRAF4
AAEL026744	0.54	0.0133333	0.0133333	0.323333	CASPS20
AAEL010270	2.98	7.47	10.3767	2.90667	CLIPC15
AAEL019781	17.4767	7.95333	50.69	20.69	CLIPE11
AAEL005792	2.99667	1.08667	7.89667	2.36	CLIPE8
AAEL019639	2.9	0.323333	0.803333	3.07667	HPX3
AAEL009474	27.5933	13.8767	164.637	26.2833	PGRPS1
AAEL009432	5.33667	0.863333	0.99	2.48667	SCRBQ3
AAEL002704	3.96667	0.256667	0.266667	2.97	SRPN23
AAEL014896	1.52667	0.14	0.226667	0.746667	TOLL9B
AAEL005293	6.38667	0.9	1.38	5.81	GALE8A
AAEL029047	7.57667	0	725.393	0.75	CECN
AAEL002601	68.7167	32.1967	78.5867	25.9333	CLIPA1
AAEL002595	201.177	97.55	127.933	48.9767	CLIPA14
AAEL015430	17.2267	10.09	10.7467	3.97667	CLIPB19
AAEL007993	0.503333	0.206667	2.83	0.126667	CLIPB27

AAEL013245	28.6767	19.7167	27.1767	10.6133	CLIPB28
AAEL005093	18.24	5.21333	30.7967	7.45333	CLIPB46
AAEL011375	0.84	0.16	0.553333	0.223333	CLIPD11
AAEL019767	24.23	8.37	27.4833	10.1767	CLIP-novel
AAEL012353	5.86333	0.876667	5.05667	1.71	CTL15
AAEL003857	18.2367	0.313333	898.757	1.6	DEFD
AAEL027792	9.46	0.246667	76.3867	0.636667	DEFE
AAEL003723	19.67	6.64	346.097	4.93	LYSC11
AAEL001794	36.02	9.76	54.2533	10.45	TEP20
AAEL008607	29.03	11.09	32.1267	12.1067	TEP3
AAEL014078	109.483	61.15	96.68	38.9733	SRPN2
AAEL008404	0.67	0.283333	1.28	0.356667	CLIPA16
AAEL005431	24.4867	15.6833	40.0433	10.9233	CLIPB37
AAEL003632	5.03333	2.94333	5.76667	2.48667	CLIPB39
AAEL012711	3.93333	3.7	2.40333	4.98333	CLIPC12
AAEL000238	0.31	0.0866667	0.593333	0.156667	CLIPD9
AAEL005641	45.4033	43.7833	115.51	22.6167	CTLGA5
AAEL014382	64.4333	66.2367	246.42	45.0633	CTLMA14
