# Supplementary material for: Ecdysone signaling mediates the trade-off between immunity and reproduction via suppression of amyloids in the mosquito Aedes aegypti
Source: PLoS Pathog. 2022 Sep 22;18(9):e1010837. doi: 10.1371/journal.ppat.1010837 (PMC9531809; doi:10.1371/journal.ppat.1010837)
Supplement: S3 Table — (PDF) [file ppat.1010837.s010.pdf]

**S3Table. Differentially expressed IMRGs in Pirk-like$^{-/-}$ and WT mosquito fat bodies with *E. cloacae* infection.** Genes with a minimum fold change of 2-fold and a false discovery rate-adjusted p-value (q-value) of < 0.05 were considered differentially expressed.

| GeneID | Pirk-like$^{-/-}$_Ec12h | Pirk-like$^{-/-}$_Ec24h | Pirk-like$^{-/-}$_PBS12h | Pirk-like$^{-/-}$_PBS24h | WT_Ec12h | WT_Ec24h | WT_PBS12h | WT_PBS24h | GeneName |
|---|---|---|---|---|---|---|---|---|---|
| AAEL002585 | 90.7867 | 185.073 | 81.03 | 127.97 | 30.2167 | 64.1067 | 15.1533 | 29.1867 | CLIPA11 |
| AAEL003253 | 11.6867 | 11.5233 | 25.2133 | 6.28667 | 9.35 | 11.1333 | 4.71333 | 15.5133 | CLIPB13B |
| AAEL014349 | 55.0733 | 171.327 | 39.4667 | 51.74 | 21.4933 | 20.5567 | 10.81 | 12.7 | CLIPB15 |
| AAEL006674 | 72.2267 | 225.323 | 57.0767 | 156.03 | 63.1733 | 212.14 | 27.94 | 187.917 | CLIPB29 |
| AAEL003632 | 4.03667 | 18.16 | 11.9667 | 7.91333 | 7.17333 | 1.87667 | 3.4 | 1.26667 | CLIPB39 |
| AAEL006168 | 87.0067 | 59.15 | 89.7867 | 52.97 | 70.6133 | 27.2533 | 35.1167 | 10.7767 | CLIPB42 |
| AAEL005093 | 39.0433 | 124.873 | 46.4633 | 55.5 | 13.5533 | 29.94 | 4.41333 | 9.58 | CLIPB46 |
| AAEL000224 | 0.153333 | 0.0766667 | 0.106667 | 0.0266667 | 2.46 | 5.25667 | 0.736667 | 7.37667 | CLIPD7 |
| AAEL014141 | 19.2467 | 35.43 | 32.0333 | 34.0767 | 23.76 | 31.9033 | 10.11 | 19.3233 | SRPN5 |
| AAEL006568 | 6.68333 | 20.8333 | 0.54 | 3.82333 | 37.4267 | 947.363 | 140.767 | 1035.73 | SRPN |
| AAEL029047 | 577.217 | 847.22 | 411.823 | 419.527 | 25.8767 | 61.7267 | 1.9 | 0.936667 | CECN |
| AAEL003857 | 1016.31 | 1132.89 | 432.797 | 1302.59 | 56.27 | 406.373 | 1.52 | 0.9 | DEFD |
| AAEL003832 | 272.027 | 156.347 | 40.6833 | 9.13333 | 16.1933 | 48.6233 | 0.726667 | 0.53 | DEFC |
| AAEL027792 | 94.3 | 164.06 | 71.43 | 127.217 | 3.79333 | 200.563 | 1.84333 | 0.163333 | DEFE |
| AAEL005641 | 211.58 | 753.74 | 217.437 | 182.887 | 34.6333 | 118.55 | 6.09667 | 2.07667 | CTLGA5 |
| AAEL011621 | 3.69333 | 5.74 | 4.85667 | 2.42667 | 1.84667 | 1.75 | 0.926667 | 1.48667 | CTLMA13 |
| AAEL019633 | 7.12333 | 5.31333 | 3.47667 | 3.57 | 1.91 | 1.60333 | 0.87 | 0.99 | CTLGA9 |
| AAEL002524 | 11.0733 | 12.4633 | 6.97667 | 8.33 | 2.96 | 15.4933 | 1.44333 | 4.09667 | CTL24 |
| AAEL000563 | 3.79667 | 1.74 | 3.65 | 2.86 | 6.26667 | 7.86 | 2.51667 | 7.67667 | CTLMA15 |
| AAEL015404 | 57.2533 | 98.2667 | 105.79 | 37.2167 | 41.5733 | 27.23 | 15.92 | 24.8167 | LYSC7B |

| | | | | | | | | | |
|---|---|---|---|---|---|---|---|---|---|
| AAEL015404 | 57.2533 | 98.2667 | 105.79 | 37.2167 | 41.5733 | 27.23 | 15.92 | 24.8167 | LYSC7A |
| AAEL009474 | 173.01 | 175.823 | 60.7233 | 99.69 | 37.9667 | 38.4833 | 14.6133 | 7.31333 | PGRPS1 |
| AAEL010171 | 7.18 | 65.3367 | 3.04333 | 9.79333 | 1.8 | 3.80333 | 0.83 | 1.40333 | PGRPLB |
| AAEL014896 | 0.38 | 0.396667 | 0.31 | 1.08667 | 0.706667 | 0.3 | 0.166667 | 0.493333 | TOLL9B |
| AAEL015019 | 1.14333 | 2.68667 | 1.65333 | 0.95 | 2.50333 | 10.55 | 1.12 | 3.42 | TOLL4 |
| AAEL026478 | 0.463333 | 0.81 | 0.556667 | 0.92 | 1.23 | 1.90333 | 2.81667 | 1.02 | TOLL2 |
| AAEL011009 | 1.34667 | 2.06667 | 1.01667 | 0.713333 | 1.66667 | 0.85 | 0.753333 | 0.753333 | FREP13 |
| AAEL006704 | 466.607 | 927.803 | 78.7533 | 89.0133 | 123.183 | 1.28 | 2.51 | 0.166667 | FREP18 |
| AAEL019868 | 21.2433 | 32.85 | 14.11 | 13.1 | 7.48667 | 10.2767 | 2.15333 | 1.95333 | FREP17 |
| AAEL011009 | 1.34667 | 2.06667 | 1.01667 | 0.713333 | 1.66667 | 0.85 | 0.753333 | 0.753333 | FREP8 |
