# Supplementary material for: Ecdysone signaling mediates the trade-off between immunity and reproduction via suppression of amyloids in the mosquito Aedes aegypti
Source: PLoS Pathog. 2022 Sep 22;18(9):e1010837. doi: 10.1371/journal.ppat.1010837 (PMC9531809; doi:10.1371/journal.ppat.1010837)
Supplement: Attachment — Submitted filename: PPATHOGENS-D-22-00692 Comments.pdf [file ppat.1010837.s011.pdf]

Immunity and reproduction are two important processes in insects that compete for limited resources. A delicate balance between immunity and reproduction is needed for survival and propagation. While some studies have indicated that insect hormones are implicated in the trade-off, the actual hormone-regulated effector molecules have not been identified. Wang et al. reported in this study that Pirk-like, a negative regulator of the IMD pathway, is upregulated by 20-hydroxyecdysone (20E) after a blood meal in the yellow fever mosquito *Aedes aegypti* to attenuate mosquito innate immunity in favor of egg maturation. The authors provide clear evidence showing that the 20E receptor complex directly binds to the promoter of the Pirk-like gene to increase its expression after a blood meal. Most importantly, Pirk-like was able to directly interact with PGRP-LC, PGRP-LE and IMD. Knockout of Pirk-like enhanced mosquito survival after bacterial infection and this enhancement was nullified by the treatment of Thioflavin T, an inhibitor of amyloid formation, suggesting that Pirk-like represses the formation of functional IMD amyloid fibril which is required for the signal transduction of IMD pathway. This study addresses a fundamental question in mosquito biology and provides a new target that may be exploited for the control of mosquito-borne diseases. The results are supported by substantial evidence and statistical analysis. The manuscript is well written and is comprehensible to the general reader. I have several comments that may help the authors to improve this manuscript.

Specific points:

1. In addition to the IMD pathway, Pirk-like also downregulates genes related to ribosome, lysosome, synaptic vesicle cycle, and amino sugar and nucleotide sugar metabolism. What could be the potential mechanism? Will these downregulations affect innate immunity and egg maturation?
2. Lines 55-57. Only the first gonadotrophic cycle consists of a post-eclosion phase (PE) and a post blood meal phase (PBM).
3. Lines 175-176. Pirk-like was induced by 20E in EcR- and USP-overexpressed Ae. aegypti (Aag2) cells.
4. Line 188. The assay coupled with ChIP seems to be quantitative PCR or real-time PCR, not reverse-transcription PCR.
5. Lines 196-197. The extra band could come from (or result from) endogenous EcR and USP. It is not interfered with by endogenous proteins.
6. Lines 222-223. The sequence similarity, not variance, suggested that amyloidal aggregates could be formed in Ae. aegypti.
7. Fig. 6. The Pirk-like-/- mosquitoes induced much higher expression of PGRP-LC and Rel2 after infection with E. cloacae after a blood meal. Will Pirk-like affect immune responses in the PE phase?
8. Line 312. Vg proteins are deposited in maturing oocytes.
9. Fig. 7A. The effect of Pirk-like knockout on ovary development should also be examined before a blood meal.
10. Fig. 7D. This experiment should also include infection with E. cloacae as shown in Fig. 7B and C.
11. Line 355. It is 20E, not EcR, that blocks certain physiological processes.
12. Line 819-821. Was the differential expression compared with the levels of iEGFP-PBS?
