# Supplementary material for: Ecdysone signaling mediates the trade-off between immunity and reproduction via suppression of amyloids in the mosquito Aedes aegypti
Source: PLoS Pathog. 2022 Sep 22;18(9):e1010837. doi: 10.1371/journal.ppat.1010837 (PMC9531809; doi:10.1371/journal.ppat.1010837)
Supplement: Attachment — Submitted filename: Review response-a.docx [file ppat.1010837.s012.docx]

July 24, 2022

*PLoS Pathogens*

Dear Editor,

Thank you very much for your consideration and help. We appreciate yours and reviewer’s constructive comments. All comments are valuable and very helpful for improving this manuscript. We considered all the comments carefully, and have supplemented some experiments and revised the manuscript according to these suggestions. We hope the new version of our manuscript is now appropriately suited for publication in *PLoS Pathogens*. Below, please find our detailed point-by-point response.

Reviewer #1:

1. In addition to the IMD pathway, Pirk-like also downregulates genes related to ribosome, lysosome, synaptic vesicle cycle, and amino sugar and nucleotide sugar metabolism. What could be the potential mechanism? Will these downregulations affect innate immunity and egg maturation?

**Response: Thanks to the reviewer’s comments. The regulatory mechanism of Pirk-like on these pathways in *Aedes aegypti* is not very clear, and it may be caused by the inhibition of functional amyloid fibril formation. Several reports suggested that lysosomes are involved in amyloid degradation [1]. We tentatively speculate that the upregulation of lysosomal pathway genes in Pirk-like mutants aimed to clear amyloid and prevent its aggregation from affecting cellular function. In addition, lysosomes are involved in the cessation of vitellogenin secretion in *Ae*. *aegypti* [2]. The relationship between these pathways and innate immunity and egg maturation is unknown and requires further investigation.**

1.	Boeynaems S, Alberti S, Fawzi NL, Mittag T, Polymenidou M, Rousseau F, et al. Protein Phase Separation: A New Phase in Cell Biology. Trends Cell Biol. 2018;28(6):420-35. doi: 10.1016/j.tcb.2018.02.004. PubMed PMID: 29602697; PubMed Central PMCID: PMCPMC6034118.

2.	Raikhel AS. Lysosomes in the cessation of vitellogenin secretion by the mosquito fat body; selective degradation of Golgi complexes and secretory granules. Tissue & cell. 1986;18(1):125-42. Epub 1986/01/01. doi: 10.1016/0040-8166(86)90012-1. PubMed PMID: 3961792.

2. Lines 55-57. Only the first gonadotrophic cycle consists of a post-eclosion phase (PE) and a post blood meal phase (PBM).

**Response: Thanks for the reviewer’s suggestions. We have changed “each gonadotrophic cycle consists of a post-eclosion phase (PE) in the first cycle and a post blood meal phase (PBM) that are regulated by two major insect hormones, juvenile hormone (JH) and 20-hydroxyecdysone (20E), respectively” to “gonadotrophic cycle consists of a post-eclosion phase (PE) in the first cycle and a post blood meal phase (PBM) that are regulated by two major insect hormones, juvenile hormone (JH) and 20-hydroxyecdysone (20E), respectively”.**

3. Lines 175-176. Pirk-like was induced by 20E in EcR- and USP-overexpressed Ae. aegypti (Aag2) cells.

**Response: We agree with the reviewer that Pirk-like was induced by 20E in EcR- and USP-overexpressed *Ae. aegypti* (Aag2) cells. We have corrected it in the manuscript.**

4. Line 188. The assay coupled with ChIP seems to be quantitative PCR or real-time PCR, not reverse-transcription PCR.

**Response: Thanks for the reviewer’s suggestions, and we have changed “reverse-transcription PCR” to “quantitative PCR”.**

5. Lines 196-197. The extra band could come from (or result from) endogenous EcR and USP. It is not interfered with by endogenous proteins.

**Response: We agree with the reviewer and have changed “be interfered by” to “come from”.**

6. Lines 222-223. The sequence similarity, not variance, suggested that amyloidal aggregates could be formed in Ae. aegypti.

**Response: Thanks for the suggestions. We have changed “variance” to “similarity”.**

7. Fig. 6. The Pirk-like-/- mosquitoes induced much higher expression of PGRP-LC and Rel2 after infection with E. cloacae after a blood meal. Will Pirk-like affect immune responses in the PE phase?

**Response: We thank the reviewer for this insightful comment. We have added the experiments to analyze the expression of AMP in PE phase by qRT-PCR (Fig S6I). As shown in Fig S6I, during PE phase, only the expression of *CecB* was elevated in Pirk-like^-/-^ mosquitoes after PBS treatment, and the transcriptions of *CecB, CecG, DefD* and *GAM* were significantly higher in Pirk-like mosquitoes than in WT mosquitoes after infection.**

8. Line 312. Vg proteins are deposited in maturing oocytes.

**Response: “Some females had one ovary completely underdeveloped and had impaired Vg deposition in the ovaries” was changed to “Some females had one side ovary completely underdeveloped”.**

9. Fig. 7A. The effect of Pirk-like knockout on ovary development should also be examined before a blood meal.

**Response: Thanks for the reviewer’s proposal. We have performed a new experiment to study the effect of Pirk-like knockout on ovary development before a blood meal. As shown in Fig S7C, ovary development shows no difference between WT and Pirk-like^-/-^ mosquitoes in PE phase.**

10. Fig. 7D. This experiment should also include infection with E. cloacae as shown in Fig. 7B and C.

Response: **Thanks for the reviewer’s suggestions. We have added a new experiment. The result was showed in Fig 7D.**

11. Line 355. It is 20E, not EcR, that blocks certain physiological processes.

Response: **We agree with the reviewer and have changed “EcR” to “20E”.**

12. Line 819-821. Was the differential expression compared with the levels of iEGFP-PBS?

**Response: Yes,** **the data were normalized to the expression level of iEGFP-PBS.**

Reviewer #2: The manuscript provides a set of solid data and the experiments are presented in a logical sequence, with the following shortcomings that remain to be addressed.

1. In Abstract, Line 27-20. The two sentences are confusing. “*Drosophila melanogaster* Pirk, which a negative regulator of the IMD pathway. CRISPR/Cas9 knockout of Pirk-like has shown that it represses the IMD pathway.”

**Response: Thanks for the reviewer’s suggestions. In the abstract, we wanted to show that mosquito Pirk-like is an ortholog of *Drosophila melanogaster* Pirk, and knockout of Pirk-like represses the IMD pathway. We have revised the abstract.**

**2.** Figure 5, amyloid formation in the fat body of iEcR mosquitoes should be detected to further support the conclusion that amyloid was closely associated with 20E.

**Response: Thanks for the suggestions. We have performed a new experiment to detect the amyloid formation in the fat body od iECR mosquitoes. The result was showed in Fig S5E.**

3. Line 41. Replace “20-hydroxyecdysone” by “20-hydroxyecdysone (20E)”.

**Response: Thanks for the reviewer’s suggestions. We have replaced “20-hydroxyecdysone” with “20-hydroxyecdysone (20E)”.**

4. Line 92. “Aedes. Aegypti” should be “Aedes aegypti”.

**Response: Thanks for the suggestions. We have changed “*Aedes. Aegypti*” to “*Aedes aegypti*”.**

5. Line 279. “than did WT_Ec mosquitoes or normal controls” is incorrect.

**Response: Thanks for the reviewer’s suggestions. We have changed “than did WT_Ec mosquitoes or normal controls” to “than WT_Ec mosquitoes or normal controls”.**

6. Line 293. “is likely a direct Rel2 transcriptional target” should be “is likely a direct transcriptional target of Rel2”.

**Response: Thanks for the reviewer’s suggestions. We have changed “is likely a direct Rel2 transcriptional target” to “is likely a direct transcriptional target of Rel2”.**

7. Line 415. “LB medium no antibiotics” should be “LB medium without antibiotics”.

**Response: Thanks for the reviewer’s suggestions. We have changed “no” to “without”.**

Reviewer #3: The only concern regarding the manuscript that I believe would improve the manuscript refers to better show the trade off between immunity and reproduction that derives from the experimental approach followed by the authors, that is, the elimination of EcR during the first gonadotrophic cycle of Aedes. It would be interesting that the authors show the effect of eliminating EcR on reproductive parameters and on the response to Enterobacter cloacae infection. That is, as they do with the Pirk-like mutant, it would be interesting to measure ovarian growth, number of eggs deposited and vitellogenin production in EcR-depleted females with and without Enterobacter infection compared to wild type females in order to check the effect of the absence of EcR on the control of both processes.

**Response: Thanks for the reviewer’s comments.** **We have performed the new experiments to verify the effect of EcR silencing on mosquito egg production and Vg expression with or without Enterobacter infection. Consistent to previous results, the egg production of iEcR_Ec and iEGFP_Ec mosquitoes was significantly reduced compared to control sample (S7G Fig). Accordingly, reduced Vg level in iEcR_Ec mosquitoes was observed (S7H Fig).**
