# Supplementary material for: Ecdysone signaling mediates the trade-off between immunity and reproduction via suppression of amyloids in the mosquito Aedes aegypti
Source: PLoS Pathog. 2022 Sep 22;18(9):e1010837. doi: 10.1371/journal.ppat.1010837 (PMC9531809; doi:10.1371/journal.ppat.1010837)
Supplement: Peer review history

# Peer review: Decision Letter 0

**DOI:** 10.1371/journal.ppat.1010837.r001

**Authors:** Kirk W. Deitsch, Kenneth D Vernick

1 Jun 2022

Dear Dr. Zou,

Thank you very much for submitting your manuscript "Ecdysone signaling mediates the trade-off between immunity and reproduction via suppression of amyloids in the mosquito Aedes aegypti" for consideration at PLOS Pathogens. As with all papers reviewed by the journal, your manuscript was reviewed by members of the editorial board and by several independent reviewers. The reviewers appreciated the attention to an important topic. Based on the reviews, we are likely to accept this manuscript for publication, providing that you modify the manuscript according to the review recommendations.

The reviewers made several suggestions for improving the manuscript. However, the performance of any new experimental work is at the discretion of the authors, and is not required for the revision, which can be accomplished by revision of the text.

Please prepare and submit your revised manuscript within 30 days. If you anticipate any delay, please let us know the expected resubmission date by replying to this email.

When you are ready to resubmit, please upload the following:

[1] A letter containing a detailed list of your responses to all review comments, and a description of the changes you have made in the manuscript.

Please note while forming your response, if your article is accepted, you may have the opportunity to make the peer review history publicly available. The record will include editor decision letters (with reviews) and your responses to reviewer comments. If eligible, we will contact you to opt in or out

[2] Two versions of the revised manuscript: one with either highlights or tracked changes denoting where the text has been changed; the other a clean version (uploaded as the manuscript file).

Important additional instructions are given below your reviewer comments.

Thank you again for your submission to our journal. We hope that our editorial process has been constructive so far, and we welcome your feedback at any time. Please don't hesitate to contact us if you have any questions or comments.

Sincerely,

Kenneth D Vernick

Associate Editor

PLOS Pathogens

Kirk Deitsch

Section Editor

PLOS Pathogens

Kasturi Haldar

Editor-in-Chief

PLOS Pathogens

​orcid.org/0000-0001-5065-158X

Michael Malim

Editor-in-Chief

PLOS Pathogens

orcid.org/0000-0002-7699-2064

***********************

Performance of any new experimental work is at the discretion of the authors, and is not required for the revision, which can be accomplished by revision of the text.

Reviewer Comments (if any, and for reference):

Reviewer's Responses to Questions

**Part I - Summary**

Please use this section to discuss strengths/weaknesses of study, novelty/significance, general execution and scholarship.

Reviewer #1: Immunity and reproduction are two important processes in insects that compete for limited resources. A delicate balance between immunity and reproduction is needed for survival and propagation. While some studies have indicated that insect hormones are implicated in the trade-off, the actual hormoneregulated effector molecules have not been identified. Wang et al. reported in this study that Pirk-like, a negative regulator of the IMD pathway, is upregulated by 20-hydroxyecdysone (20E) after a blood meal in the yellow fever mosquito Aedes aegypti to attenuate mosquito innate immunity in favor of egg maturation. The authors provide clear evidence showing that the 20E receptor complex directly binds to the promoter of the Pirk-like gene to increase its expression after a blood meal. Most importantly, Pirklike was able to directly interact with PGRP-LC, PGRP-LE and IMD. Knockout of Pirk-like enhanced mosquito survival after bacterial infection and this enhancement was nullified by the treatment of Thioflavin T, an inhibitor of amyloid formation, suggesting that Pirk-like represses the formation of functional IMD amyloid fibril which is required for the signal transduction of IMD pathway. This study addresses a fundamental question in mosquito biology and provides a new target that may be exploited for the control of mosquito-borne diseases. The results are supported by substantial evidence and statistical analysis. The manuscript is well written and is comprehensible to the general reader. 

Reviewer #2: This article revealed that insect hormone 20E and its receptor EcR coordinate the activity of the immune pathway, IMD after blood meals. It highlights the role of 20E on maintaining a delicate balance between reproduction and immunity. It demonstrated that the expression of Pirk-like was mainly determined by 20E during vitellogenesis. Pirk-like terminated amyloid formation, which is mediated by PGRP-LC, PGRP-LE and IMD. And it suppressed the downstream IMD signaling and helped female mosquito fertility. Knockout of Pirk-like in mosquitoes led to more resistance to bacterial infection, along with impaired reproduction. A novel aspect is the demonstration that 20E could modulate functional amyloid formation in mosquitoes. The findings presented in this work revealed a mechanism by which the anautogenous mosquitoes inhibit immune activation during blood meals. 

Reviewer #3: The manuscript by Wang et al. is a very interesting study addressing the trade-off balance between immunity and reproduction in adult female mosquito Aedes aegypti. The authors addresses this topic by analyzing the role of the main gonadotrophic hormone, 20-hydroxyecdysone (20E) and its receptor EcR in the regulation of the immune deficiency (IMD) pathway and the oocyte development during the reproductive phase of the mosquito, that is the post blood meal phase. By using a combination of RNAi, CRISPR-Cas9, and several genomic and molecular biology techniques, the authors clearly show that 20E and EcR are required to downregulate the IMD pathway by directly inducing the expression of Pirk-like, a negative regulator of the IMD pathway. In addition, the authors reveal that this effect is mediated by the Pirk-like-mediated impairment of the formation of amyloid aggregates. In summary, the authors clearly show that the dual role of 20E and EcR in inducing proper oogenesis and repressing innate immunity at the same time is critical for mosquito fertility.

The study is thorough, very well executed and the conclusions are properly supported by the data presented. It is relevant to note that the information reported is very interesting contribution to the understanding of the molecular basis of the trade-off between immunity and reproduction in adult insects, which in the case of mosquitoes is of paramount importance given its relevance in the transmission of deadly pathogens. In summary, the work is scientifically very sound and worth it for publication.

**********

**Part II – Major Issues: Key Experiments Required for Acceptance**

Please use this section to detail the key new experiments or modifications of existing experiments that should be absolutely required to validate study conclusions.

Generally, there should be no more than 3 such required experiments or major modifications for a "Major Revision" recommendation. If more than 3 experiments are necessary to validate the study conclusions, then you are encouraged to recommend "Reject".

Reviewer #1: (No Response)

Reviewer #2: (No Response)

Reviewer #3: (No Response)

**********

**Part III – Minor Issues: Editorial and Data Presentation Modifications**

Please use this section for editorial suggestions as well as relatively minor modifications of existing data that would enhance clarity.

Reviewer #1: I have several comments that may help the authors to improve this manuscript.

Specific points:

1. In addition to the IMD pathway, Pirk-like also downregulates genes related to ribosome, lysosome, synaptic vesicle cycle, and amino sugar and nucleotide sugar metabolism. What could be the potential mechanism? Will these downregulations affect innate immunity and egg maturation?

2. Lines 55-57. Only the first gonadotrophic cycle consists of a post-eclosion phase (PE) and a post blood meal phase (PBM).

3. Lines 175-176. Pirk-like was induced by 20E in EcR- and USP-overexpressed Ae. aegypti (Aag2) cells.

4. Line 188. The assay coupled with ChIP seems to be quantitative PCR or real-time PCR, not reverse-transcription PCR.

5. Lines 196-197. The extra band could come from (or result from) endogenous EcR and USP. It is not interfered with by endogenous proteins.

6. Lines 222-223. The sequence similarity, not variance, suggested that amyloidal aggregates could be formed in Ae. aegypti.

7. Fig. 6. The Pirk-like-/- mosquitoes induced much higher expression of PGRP-LC and Rel2 after infection with E. cloacae after a blood meal. Will Pirk-like affect immune responses in the PE phase?

8. Line 312. Vg proteins are deposited in maturing oocytes.

9. Fig. 7A. The effect of Pirk-like knockout on ovary development should also be examined before a blood meal.

10. Fig. 7D. This experiment should also include infection with E. cloacae as shown in Fig. 7B and C.

11. Line 355. It is 20E, not EcR, that blocks certain physiological processes.

12. Line 819-821. Was the differential expression compared with the levels of iEGFP-PBS?

Reviewer #2: The manuscript provides a set of solid data and the experiments are presented in a logical sequence, with the following shortcomings that remain to be addressed.

Specific comments:

1. In Abstract, Line 27-20. The two sentences are confusing. “Drosophila melanogaster Pirk, which a negative regulator of the IMD pathway. CRISPR/Cas9 knockout of Pirk-like has shown that it represses the IMD pathway.”

2. Figure 5, amyloid formation in the fat body of iEcR mosquitoes should be detected to further support the conclusion that amyloid was closely associated with 20E.

3. Line 41. Replace “20-hydroxyecdysone” by “20-hydroxyecdysone (20E)”.

4. Line 92. “Aedes. Aegypti” should be “Aedes aegypti”.

5. Line 279. “than did WT_Ec mosquitoes or normal controls” is incorrect.

6. Line 293. “is likely a direct Rel2 transcriptional target” should be “is likely a direct transcriptional target of Rel2”.

7. Line 415. “LB medium no antibiotics” should be “LB medium without antibiotics”.

Reviewer #3: The only concern regarding the manuscript that I believe would improve the manuscript refers to better show the trade off between immunity and reproduction that derives from the experimental approach followed by the authors, that is, the elimination of EcR during the first gonadotrophic cycle of Aedes. It would be interesting that the authors show the effect of eliminating EcR on reproductive parameters and on the response to Enterobacter cloacae infection. That is, as they do with the Pirk-like mutant, it would be interesting to measure ovarian growth, number of eggs deposited and vitellogenin production in EcR-depleted females with and without Enterobacter infection compared to wild type females in order to check the effect of the absence of EcR on the control of both processes.

**********

PLOS authors have the option to publish the peer review history of their article (what does this mean?). If published, this will include your full peer review and any attached files.

If you choose “no”, your identity will remain anonymous but your review may still be made public.

**Do you want your identity to be public for this peer review?** For information about this choice, including consent withdrawal, please see our Privacy Policy.

Reviewer #1: No

Reviewer #2: No

Reviewer #3: No

Figure Files:

While revising your submission, please upload your figure files to the Preflight Analysis and Conversion Engine (PACE) digital diagnostic tool, https://pacev2.apexcovantage.com. PACE helps ensure that figures meet PLOS requirements. To use PACE, you must first register as a user. Then, login and navigate to the UPLOAD tab, where you will find detailed instructions on how to use the tool. If you encounter any issues or have any questions when using PACE, please email us at figures@plos.org.

Data Requirements:

Please note that, as a condition of publication, PLOS' data policy requires that you make available all data used to draw the conclusions outlined in your manuscript. Data must be deposited in an appropriate repository, included within the body of the manuscript, or uploaded as supporting information. This includes all numerical values that were used to generate graphs, histograms etc.. For an example see here: http://www.plosbiology.org/article/info%3Adoi%2F10.1371%2Fjournal.pbio.1001908#s5.

Reproducibility:

To enhance the reproducibility of your results, we recommend that you deposit your laboratory protocols in protocols.io, where a protocol can be assigned its own identifier (DOI) such that it can be cited independently in the future. Additionally, PLOS ONE offers an option to publish peer-reviewed clinical study protocols. Read more information on sharing protocols at https://plos.org/protocols?utm_medium=editorial-email&utm_source=authorletters&utm_campaign=protocols

References:

Please review your reference list to ensure that it is complete and correct. If you have cited papers that have been retracted, please include the rationale for doing so in the manuscript text, or remove these references and replace them with relevant current references. Any changes to the reference list should be mentioned in the rebuttal letter that accompanies your revised manuscript. If you need to cite a retracted article, indicate the article’s retracted status in the References list and also include a citation and full reference for the retraction notice.

Attachment Submitted filename: PPATHOGENS-D-22-00692 Comments.pdf Click here for additional data file.

# Author comment: Author response to Decision Letter 0

**DOI:** 10.1371/journal.ppat.1010837.r002

2 Aug 2022

Attachment Submitted filename: Review response-a.docx Click here for additional data file.

# Editor report: Decision Letter 1

**DOI:** 10.1371/journal.ppat.1010837.r003

**Authors:** Kirk W. Deitsch, Kenneth D Vernick

29 Aug 2022

Dear Dr. Zou,

We are pleased to inform you that your manuscript 'Ecdysone signaling mediates the trade-off between immunity and reproduction via suppression of amyloids in the mosquito Aedes aegypti' has been provisionally accepted for publication in PLOS Pathogens.

Before your manuscript can be formally accepted you will need to complete some formatting changes, which you will receive in a follow up email. A member of our team will be in touch with a set of requests.

Please note that your manuscript will not be scheduled for publication until you have made the required changes, so a swift response is appreciated.

IMPORTANT: The editorial review process is now complete. PLOS will only permit corrections to spelling, formatting or significant scientific errors from this point onwards. Requests for major changes, or any which affect the scientific understanding of your work, will cause delays to the publication date of your manuscript.

Should you, your institution's press office or the journal office choose to press release your paper, you will automatically be opted out of early publication. We ask that you notify us now if you or your institution is planning to press release the article. All press must be co-ordinated with PLOS.

Thank you again for supporting Open Access publishing; we are looking forward to publishing your work in PLOS Pathogens.

Best regards,

Kenneth D Vernick

Associate Editor

PLOS Pathogens

Kirk Deitsch

Section Editor

PLOS Pathogens

Kasturi Haldar

Editor-in-Chief

PLOS Pathogens

​orcid.org/0000-0001-5065-158X

Michael Malim

Editor-in-Chief

PLOS Pathogens

orcid.org/0000-0002-7699-2064

***********************************************************

Reviewer Comments (if any, and for reference):

# Editor report: Acceptance letter

**DOI:** 10.1371/journal.ppat.1010837.r004

**Authors:** Kirk W. Deitsch, Kenneth D Vernick

19 Sep 2022

Dear Dr. Zou,

We are delighted to inform you that your manuscript, "Ecdysone signaling mediates the trade-off between immunity and reproduction via suppression of amyloids in the mosquito Aedes aegypti," has been formally accepted for publication in PLOS Pathogens.

We have now passed your article onto the PLOS Production Department who will complete the rest of the pre-publication process. All authors will receive a confirmation email upon publication.

The corresponding author will soon be receiving a typeset proof for review, to ensure errors have not been introduced during production. Please review the PDF proof of your manuscript carefully, as this is the last chance to correct any scientific or type-setting errors. Please note that major changes, or those which affect the scientific understanding of the work, will likely cause delays to the publication date of your manuscript. Note: Proofs for Front Matter articles (Pearls, Reviews, Opinions, etc...) are generated on a different schedule and may not be made available as quickly.

Soon after your final files are uploaded, the early version of your manuscript, if you opted to have an early version of your article, will be published online. The date of the early version will be your article's publication date. The final article will be published to the same URL, and all versions of the paper will be accessible to readers.

Thank you again for supporting open-access publishing; we are looking forward to publishing your work in PLOS Pathogens. 

Best regards,

Kasturi Haldar

Editor-in-Chief

PLOS Pathogens

​orcid.org/0000-0001-5065-158X

Michael Malim

Editor-in-Chief

PLOS Pathogens

orcid.org/0000-0002-7699-2064
